# **Disruption of ClC-3-mediated 2Cl** ^−^ **/H** ^**+**^ **exchange leads to behavioural deficits and thalamic atrophy**

**DOI:** 10.1038/s41598-025-19757-2

**Published:** 2025-09-29

**Authors:** Carina Balduin, Guanxiao Qi, Michael Schöneck, Verena Trinkel, Sarah Schemmert, Gustavo A. Guzman, Stefanie Bungert-Plümke, Malte Klüssendorf, Bernd Neumaier, Dirk Feldmeyer, N. Jon Shah, Tobias Stauber, Karl-Josef Langen, Raul E. Guzman, Antje Willuweit

**Affiliations:** 1 Institute of Neuroscience and Medicine, INM-4, Forschungszentrum Jülich GmbH, 52425 Jülich, Germany; 2 Institute of Neuroscience and Medicine, INM-10, Forschungszentrum Jülich GmbH, 52425 Jülich, Germany; 3 Institute of Biological Information Processing, IBI-7, Forschungszentrum Jülich GmbH, 52425 Jülich, Germany; 4 Institute of Biological Information Processing, IBI-1, Forschungszentrum Jülich GmbH, 52425 Jülich, Germany; 5 Institute for Molecular Medicine, MSH Medical School Hamburg, Hamburg, Germany; 6 Institute of Neuroscience and Medicine, INM-5, Forschungszentrum Jülich GmbH, 52425 Jülich, Germany; 7 Institute of Radiochemistry and Experimental Molecular Imaging, University Hospital Cologne and Faculty of Medicine, University of Cologne, 50937 Cologne, Germany; 8 Institute of Neuroscience and Medicine 11, INM-11, JARA, Forschungszentrum Jülich GmbH, 52425 Jülich, Germany; 9 JARA-BRAIN-Translational Medicine, Aachen, Germany; 10 Department of Neurology, RWTH Aachen University, 52062, Aachen, Germany; 11 Department of Psychiatry, Psychotherapy and Psychosomatics, RWTH Aachen University, 52062 Aachen, Germany; 12 Department of Nuclear Medicine, RWTH Aachen University, 52062 Aachen, Germany

**Keywords:** *CLCN3*, Chloride/proton exchanger, Action potentials, Thalamic neurodegeneration, Behavioural deficits, Neuroscience, Physiology

## Abstract

*CLCN3* encodes ClC-3, an endosomal 2Cl⁻/H⁺ exchanger, with pathogenic variants causing a neurodevelopmental condition marked by developmental delays, intellectual disability, seizures, hyperactivity, anxiety, and brain and retinal abnormalities. *Clcn3*^*−/−*^ mice show hippocampal and retinal degeneration, recapitulating key symptoms observed in humans. ClC-3 forms homodimers (ClC-3/ClC-3) and heterodimers with ClC-4 (ClC-3/ClC-4), with overlapping brain expression. This suggests distinct functional roles for homo- and heterodimeric assemblies and raises the question of which brain regions specifically depend on ClC-3/ClC-3 rather than ClC-3/ClC-4 complexes. Using *ex vivo* PET tracer analyses, *Clcn3*^*−/−*^ and *Clcn3*^*td/td*^ mice, we found neurodegeneration in the hippocampus and thalamus of *Clcn3*^*−/−*^, while *Clcn3*^*td/td*^ mice showed thalamic degeneration and altered neuronal excitability, including changes in action potential threshold and after hyperpolarization. *Clcn3*^*td/td*^ mice carrying a transport-deficient p.E281Q ClC-3 variant that still associates with ClC-4, thereby allowing ClC-4 to be sorted to endosomes as ClC-4/ClC-3 heterodimers, unlike in the *Clcn3*^*−/−*^ model. *Clcn3*^*td/td*^ mice also exhibited reduced weight, hyperactivity, and motor deficits, reflecting clinical features. Lower ClC-4 levels in thalamus predict a predominant thalamic expression of ClC-3/ClC-3 homodimers. Overall, our findings indicate a region-specific function of ClC-3/ClC-3 homodimeric complexes and highlight the importance of ClC-3 transport activity in thalamic neuron survival, with electrophysiological dysfunction likely contributing to neurodegeneration.

**Supplementary Information:**

The online version contains supplementary material available at 10.1038/s41598-025-19757-2.

## Introduction

Defective endo-lysosomal function is a critical causal factor for neuronal death in several neurodegenerative disorders^1–4^. Non-proliferating neurons are by far the most susceptible to endo-lysosomal system dysfunction. Any disruption along the endo-lysosomal pathway can perturb crucial processes such as protein trafficking, sorting, recycling, and degradation, ultimately culminating in neuronal dysfunction and cell death^2^. The endo-lysosomal system encompasses early endosomes, recycling endosomes, late endosomes, and lysosomes, maintaining a luminal pH range of 6.5 to 4.5^5^. The concerted action of the ATP-dependent proton pump serves as the primary driver for proton transport within endosomes^5^, with key factors such as proton leaks, ion channels and transporters being essential for regulating and maintaining the internal pH in these organelles^6,7^. Thus, pH regulation, particularly mediated by associated channels and transporters, emerges as critical for the proper functioning of the endo-lysosomal system, thereby significantly influencing neuronal function and viability. The neuronal intracellular chloride/proton (2Cl^−^/H^+^) exchangers ClC-3 and ClC-4 are integral components of the endo-lysosomal pathway, residing in distinct organelles within it^8–10^. Their transport mechanism is crucial for the functionality of the endo-lysosomal network, as demonstrated by the pathological phenotypes exhibited by patients with the rare *CLCN3* and *CLCN4* conditions, or by animal models upon disruption of ClC-3^10–17^. Pathogenic *CLCN3* variants in humans result in clinical features such as intellectual disability, seizure, severe neuropsychiatric disorders, brain abnormality, and hippocampal and retinal degeneration with delayed gross and fine motor development^13,15^. The severity of clinical symptoms in patients with homozygous loss of ClC-3 resembles phenotypes observed in *Clcn3*^*−/−*^ mice^18–21^. While *Clcn4*^*−/−*^ mice show a wild-type-like phenotype^22^, disease-causing *CLCN4* variants, resulting in a complete loss of ClC-4 transport function, lead to intellectual disability, seizures, and psychiatric disorders in humans^11,12,16,23–25^. The genomic context of *CLCN4* may account for the observed difference between animal models and human clinical features. In *Mus musculus*, it is located on chromosome 7, while in humans, it resides on the X-chromosome^14,26,27^. Unlike other neuronal 2Cl^−^/H^+^ exchangers, alternative splicing of *CLCN3* generates translated variants targeted to recycling and late endosomes, as well as lysosomes^9^. Because ClC-4 lacks endosomal sorting signals^28^, trafficking from the endoplasmic reticulum (ER) to endosomes entirely relies on its ability to oligomerize with protein family members^8^. ClC-4 forms heterodimers with ClC-3 to ensure protein stability and proper trafficking to endosomes^8^. Therefore, under physiological conditions, ClC-4 is likely not present as a homomer in endosomes, but rather as a part of ClC-3/ClC-4 heterodimeric complexes^29^. Recent studies have shown that the integrity of higher brain structures, like the hippocampus and retina, is critically dependent on the presence of endosomal CLC transporters and their essential 2Cl^−^/H^+^-exchange function^21,30^. This evidence conclusively demonstrates that ClC-3 and ClC-4 function as a heterodimeric ClC-3/ClC-4 complex within the brain^21,30^. However, the neurological symptoms and severity in individuals with *CLCN3* and *CLCN4* mutations are not ubiquitous, as they only overlap to a certain extent^12,13^. This argues against a ubiquitous heterodimeric function of ClC-3/ClC-4 across the brain. We therefore hypothesize that these transporters may have brain region-specific expression profiles, leading to homodimeric or heterodimeric assemblies. Furthermore, there is currently no information available about the vulnerability of other brain regions to the endosomal dysfunction or disruption of these transporters and their associated 2Cl^−^/H^+^-exchange machinery, aside from the hippocampus and retina.

To explore these aspects, we used knock-in *Clcn3*^*td/td*^ mice with an ion transport-deficient ClC-3 mutant^21^, *Clcn3*^*−/−*^ and wild-type (WT) mice and employed the PET radiotracer *cis*-4-[^18^F]-fluoro-d-proline (*cis*-F-d-proline) to investigate neurodegenerative processes in various brain regions of these mice. *cis*-F-d-proline has been used to detect primary brain lesions and secondary neurodegeneration in remote brain regions of rodents after cortical ischemia or harbouring cerebral gliomas^31,32^. The transport-deficient knock-in *Clcn3*^*td/td*^ mouse model expresses a ClC-3 mutant that abolishes ion transport but conserves its ability to be sorted to endosomes and form heterodimers with ClC-4^21,33^. In ex vivo autoradiographic experiments, *cis-*F-d-proline depicted neurodegeneration and inflammation in the hippocampus of *Clcn3*^*−/−*^ mice and, surprisingly, in the thalamus of both *Clcn3*^*−/−*^ and *Clcn3*^*td/td*^ mice. These findings were underpinned by defective electrophysiological properties of *Clcn3*^*td/td*^ thalamic neurons. Homozygous *Clcn3*^*td/td*^ and *Clcn3*^*−/−*^ mice exhibited reduced body weight and displayed hyperactivity, as well as motor impairment in longitudinal behavioural analyses. In addition, our experiments revealed lower ClC-4 expression levels in the thalamus compared to the hippocampus in wild-type mice.

We identified a brain region that is nearly devoid of endosomal ClC-4. The distinct ClC-4 expression pattern between the thalamus and hippocampus, combined with similar ClC-3 levels in both regions, predicts the prevalence of ClC-3/ClC-3 homodimers rather than ClC-3/ClC-4 heterodimers in the thalamus of mammalian WT. Therefore, we conclude that thalamic neuronal function and survival rely on the endosomal 2Cl^−^/H^+^-exchange activity of homodimeric ClC-3 complexes. Our findings stress the importance of considering the thalamus as an essential brain region for the understanding of *CLCN3*-related conditions.

## Results

### ClC-3 chloride/proton transport activity is indispensable to maintain the viability of thalamic neurons

**Fig. 1:**
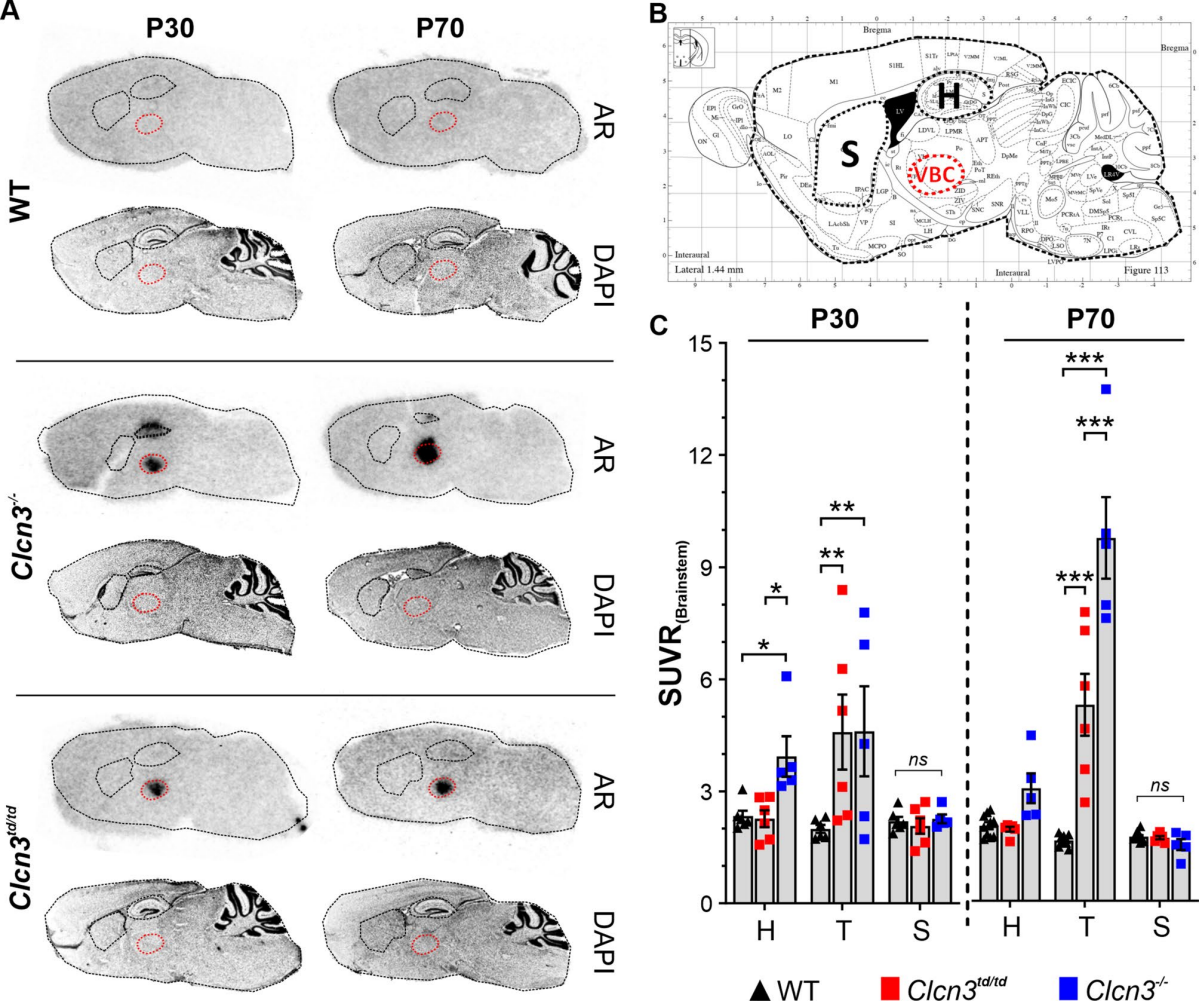
Uptake of *cis*-F-d-proline in areas of neurodegeneration in the brains of *Clcn3*^*td/td*^ and *Clcn3*^*−/−*^ mice. (**A**) Reprojection to the anatomical map identified *cis*-F-D-proline tracer accumulation in the hippocampus and ventrobasal complex (VBC) of the thalamus in *Clcn3*^*−/−*^ mice, as indicated by red ellipse. Hippocampus (H) is marked by a second ellipse above the VPM, as well as striatum (S). In *Clcn3*^*td/td*^ mice accumulation of the radiotracer was only evident in the VBC region at both ages. Adjacent to the autoradiographic slices (AR), brain slices were stained with DAPI for histological overview (**B**) Schematic overview of a sagittal section of the mouse brain atlas 1.44 mm lateral to Bregma (modified from Paxinos and Franklin^36^) used to identify neurodegeneration in brain autoradiograms of WT and *Clcn3*^*td/td*^ and *Clcn3*^*−/−*^ mice after injection of the radiotracer *cis*-4-[^18^F]-fluoro-d-proline (*cis*-F-d-proline). (**C**) *cis*-F-d-proline tracer uptake in the thalamus of *Clcn3*^*td/td*^ (red squares) and *Clcn3*^*−/−*^ (blue squares) mice was significantly higher for P30 mice and P70 mice compared to WT (black up triangles), as illustrated by the standardized uptake value ratio (SUVR_(Brainstem)_). Statistical calculations were conducted by two-way ANOVA with Fisher post hoc test (SUVR_(Brainstem)_ ± SEM, *n* = 5 to 10 animals per group, 6 slices per animal for hippocampus and striatum, 3 slices per animal for thalamus). Significant data are marked by asterisks. *P ≤ 0.05**P ≤ 0.01 ***P ≤ 0.0001. H, hippocampus; S, striatum; T, thalamus.

Neurodegeneration is a common feature observed in most of the CLC 2Cl^−^/H^+^ exchanger knockout mouse models^18–20,34,35^, emphasizing how crucial these proteins are for the homeostatic control of brain function. We used *Clcn3*^*−/−*^ mice to explore which brain regions may be affected beyond the hippocampus and, thus, are physiologically dependent on the 2Cl^−^/H^+^ exchange function. *cis*-F-d-proline, a high-sensitivity PET radiotracer used to detect primary and secondary neurodegenerative processes in the brain^32^, was injected *i.v.* into P30 and P70 *Clcn3*^*−/−*^ mice, and *ex vivo* autoradiography of the brain was performed to visualize neuronal damage (Fig. 1A). Also, *cis*-F-d-proline uptake in the brains was quantified as the standardized uptake value ratio normalized to the brainstem (SUVR_(Brainstem)_) for several brain regions (Table 1).

**Table 1:** *cis*-4-[^18^F]-fluoro-d-proline in the different brain areas of *Clcn3*^*td/td*^ and *Clcn3*^*−/−*^ mice.

	Brain area	*cis*-4-[^18^F]-fluoro-d-proline			
		SUVR_(Brainstem)_ P30	Statistics	SUVR_(Brainstem)_ P70	Statistics
*Clcn3*^*td/td*^	Hippocampus	2.26 ± 0.22	*n.s*	1.99 ± 0.07	*n.s*
	Thalamus	4.65 ± 1.01	p < .01	5.45 ± 0.86	p < .0001
	Striatum	2.07 ± 0.21	*n.s*	1.76 ± 0.05	*n.s*
*Clcn3*^*−/−*^	Hippocampus	3.93 ± 0.54	p < .05	3.13 ± 0.41	*n.s*
	Thalamus	4.61 ± 1.21	p < .01	10.09 ± 1.13	p < .0001
	Striatum	2.26 ± 0.12	*n.s*	1.56 ± 0.15	*n.s*
WT	Hippocampus	2.33 ± 0.15		2.09 ± 0.09	
	Thalamus	1.99 ± 0.11		1.68 ± 0.05	
	Striatum	2.19 ± 0.12		1.79 ± 0.04	

P(x) = postnatal age in days, SUVR_(Brainstem)_ = standardized uptake value ratio normalized to brainstem.

Data are presented as mean ± SEM. Statistics calculated with: two-way ANOVA, Fisher post hoc analysis, WT *versus* *Clcn3*^td/td^ or *Clcn3*^−/−^.

*n.s.* not significant.

**Fig. 2:**
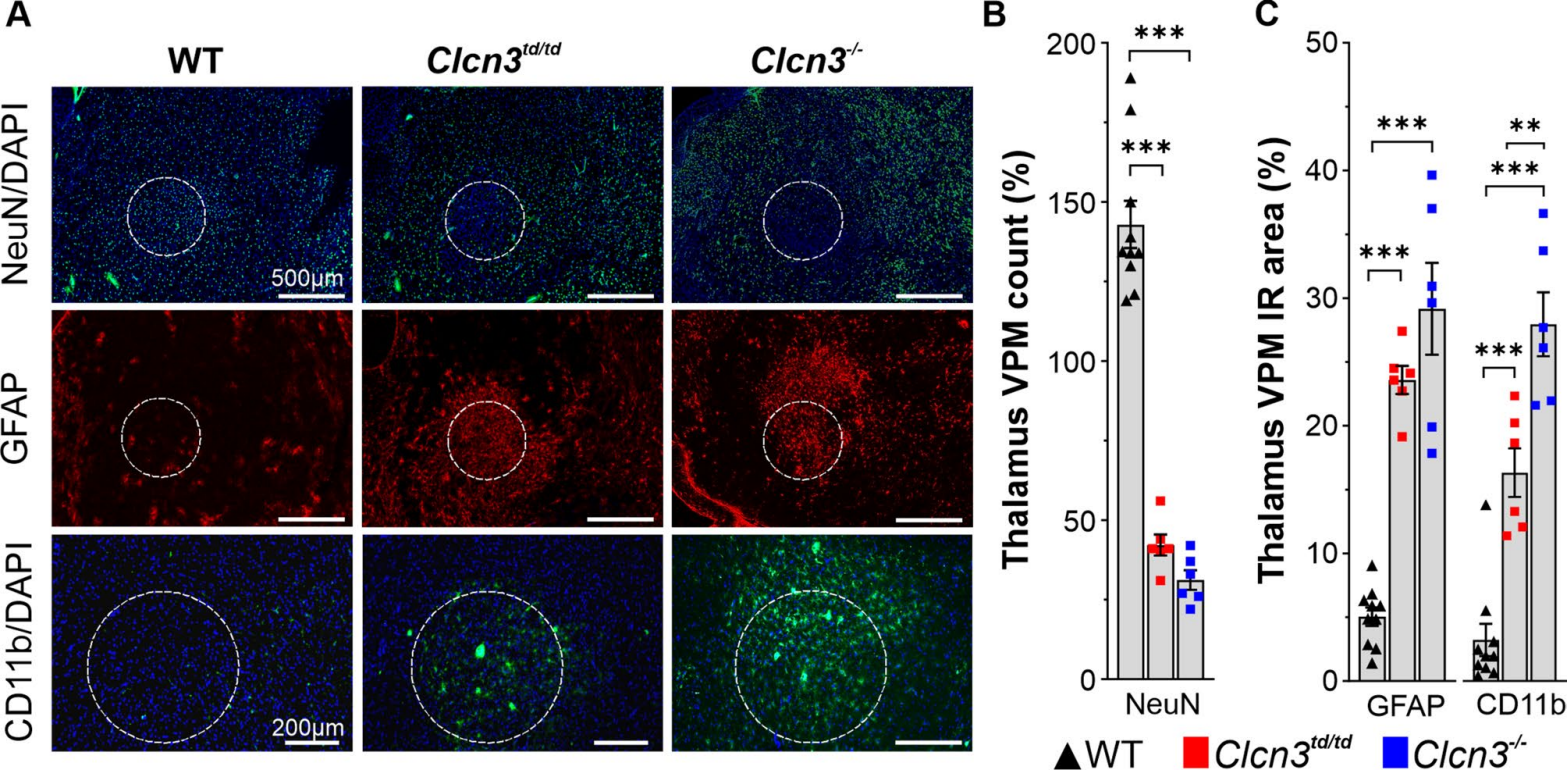
Loss of NeuN labelled cells and gliosis in the thalamic ventral posteromedial nucleus (VPM) region of *Clcn3*^*td/td*^ and *Clcn3*^*−/−*^ mice. (**A**) Representative immunofluorescence images of thalamus in sagittal sections of WT, *Clcn3*^*−/−*^ and *Clcn3*^*td/td*^ mice at P70 stained against neurons (NeuN, green), activated astrocytes (GFAP, red) and reactive microglia (CD11b, green). Cell nuclei were counterstained with DAPI (blue). (**B**) Quantification in the VPM as part of the ventrobasal complex (VBC) of the thalamus indicates a significant lower number of NeuN-positive neurons, while (**C**) percentage of immunoreactive (IR) area of activated astrocytes (GFAP) and microglia (CD11b) were significantly upregulated in this brain area for homozygous *Clcn3*^*td/td*^ (red squares) and *Clcn3*^*−/−*^ mice (blue squares) compared to WT (black up triangles). Loss of neurons is accompanied by gliosis in the thalamic VPM region of *Clcn3*^*−/−*^ and *Clcn3*^*td/td*^ mice (white dotted outline in (**A**)). Data are presented as mean ± SEM calculated from three to five slices, respectively. Statistical calculation was conducted by one-way ANOVA with Tukey´s post hoc analysis WT vs. *Clcn3*^*−/−*^ and WT vs. *Clcn3*^*td/td*^. WT *n* = 10; *Clcn3*^*td/td*^ and *Clcn3*^*−/−*^, *n* = 6. Significant data are marked by asterisks. **P ≤ 0.01; ***P ≤ *0.0001*.

At postnatal day 30 (P30), juvenile *Clcn3*^*−/−*^ mice showed significant accumulation of *cis*-F-d-proline in the hippocampus, verified by reprojection to the anatomic atlas^36^ (Fig. 1B), indicating a process of neurodegeneration and neuroinflammation (Fig. 1A and C). Mean SUVR_(Brainstem)_ values were nearly two-fold higher in *Clcn3*^*−/−*^ brains compared to WT for the respective brain region (two-way ANOVA, *P*_*P30*_ = 0.0046, *F*(2.42) = 6.137; and *P*_*P70*_ < 0.0001, F(2,54) = 91.49). This observation agrees with the described hippocampal neurodegeneration in *Clcn3*^*−/−*^^20^ and validates our approaches. *cis*-F-d-proline uptake was observed in a second brain region, identified as part of the main somatosensory nucleus complex in mammals, the ventrobasal-complex (VBC) of the thalamus, according to the anatomic atlas^36^. SUVR values in the thalamus were comparable to those in the hippocampus and significantly higher than the WT at the same age (Fig. 1A, C and Table 1). Tracer uptake in the thalamus of adult (P70) *Clcn3*^*−/−*^ mice was spatially higher compared to P30, as shown by the outlined VBC areas reprojected from adjacent histological slices. (Fig. 1B, C). At the same time, the signal in the hippocampus was lower, consistent with a reduction in hippocampal volume at this age (Suppl. Fig. 1E)^20^. We directly correlated *cis*-F-d-proline uptake on autoradiograms with neurodegeneration and inflammatory processes on immunofluorescent images in the thalamic VBC on adjacent slices, ranging from 1.84 to 1.44 mm lateral to the Bregma. Slices were stained using cell-specific antibodies for neuronal nuclei (NeuN), reactive astrocytes (GFAP), and reactive microglia (CD11b) for visualization of neuronal, inflammatory, and phagocytic cells (Fig. 2). We observed a significant reduction of about − 70% in the counts of cells positive for the neuronal marker NeuN and higher levels of immunoreactivity against GFAP and CD11b in the thalamic VBC of *Clcn3*^*−/−*^ compared to the WT mice (NeuN, WT 143 ± 7 vs *Clcn3*^*−/−*^ 31 ± 3, *P* < 0.0001; GFAP, WT 5 ± 0.7 vs *Clcn3*^*−/−*^ 29 ± 3.6, *P* < *0.0001* and CD11b, WT 3.4 ± 1.3 vs *Clcn3*^*−/−*^ 29 ± 2.6 *P* < *0.0001,* (Fig. 2A-C and Table 2), suggesting a substantial neurodegeneration accompanied by neuroinflammatory processes. In addition to the hippocampus and thalamus, other brain areas without signs of *cis*-F-d-proline accumulation, such as striatum (caudate putamen), motor cortex or brainstem, were immunostained against NeuN or GFAP for *Clcn3*^*td/td*^ and WT mice (Suppl. Figs. 1 and 2). In contrast, the striatum and hippocampus of *Clcn3*^*td/td*^ showed no obvious differences when compared to the WT (Suppl. Fig. 1G, Table 2). Thus, we concluded that the presence of ClC-3 and its associated 2Cl^*−*^/H^+^ exchange function is important for preventing neurodegeneration not only in the hippocampus but also in the thalamus of WT mice.

**Table 2:** Neurodegeneration and gliosis in the thalamic ventral posteriomedial nucleus of the thalamus (VPM) of *Clcn3*^*td/td*^ and *Clcn3*^*−/−*^ mice.

	Brain area	WT	*Clcn3*^*td/td*^	*Clcn3*^*−/−*^	Statistics
NeuN (counts)	VPM	143 ± 7	42 ± 3	31 ± 3.0	*P* < .0001, *F*(2,19) = 106.0
	Hippocampus	4855 ± 362	4778 ± 429	*n.a*	*n.s*
	Striatum	6056 ± 326	6050 ± 330	*n.a*	*n.s*
GFAP IR area (%)	VPM	5 ± 0.7	24 ± 1.1	29 ± 3.6	*P* < .0001 *F*(2,19) = 51.89
	Motor cortex	23 ± 3.6	22 ± 2.6	*n.a*	*n.s*
	Brainstem	37 ± 2.0	53 ± 5.2	*n.a*	p < .0048,
CD11b IR area (%)	VPM	3.4 ± 1.3	17 ± 2.0	29 ± 2.6	*P* < .0001, *F*(2,19) = 50.77

VPM, ventral posteromedial nucleus of the thalamus, *n.a.*, not analyzed, *n.s*., not significant.

Data are presented as mean ± SEM. Statistics: VPM: one-way ANOVA, Fisher LSD post hoc analysis WT versus *Clcn3*^*td/td*^ and *Clcn3*^*−/−*^. Motor cortex, hippocampus, striatum and brainstem: two-way repeated measures ANOVA, Fisher LSD post hoc analysis WT versus *Clcn3*^*td/td*^*.*

Given that ClC-3 and ClC-4 exhibit similar expression patterns within the mammalian brain and are known to functionally depend on each other^8,30^, it has been hypothesized that within the CNS they function as heterodimeric ClC-3/ClC-4^17^. This assumption is supported by experimental data from two independent transport-deficient mouse models showing the significance of ClC-3/ClC-4 association for the viability of neuronal cells in the hippocampus^21,30^. To verify whether this scenario also holds for thalamic neurons, we used the recently described *Clcn3*^*td/td*^ mouse model expressing an ion transport-deficient ClC-3 mutant that conserves its ability to interact with ClC-4^21^. In *Clcn3*^*td/td*^ mice, no tracer uptake could be observed in the hippocampal region at P30 or P70 (Fig. 1A, C and Table 1), which agrees with the absence of hippocampal degeneration, verified by histology (Suppl. Fig. 1)^21^. In contrast, we observed significant tracer accumulation in the thalamus of *Clcn3*^*td/td*^ mice, resembling that of the *Clcn3*^*−/−*^ at P30 (Fig. 1A, 1C and Table 1). Additionally, *Clcn3*^*td/td*^ thalamus showed no major changes in the uptake levels of *cis*-F-d-proline at P70, compared to P30 (Fig. 1B, 1C and Table 1). Overall, SUVR values differed significantly between all genotypes (two way ANOVA, *P*_*P30*_ < 0.05, F(2.42) = 5.009 and *P*_*P70* _< 0.0001, F(2,54) = 48.33) and an overall significant interaction between the brain regions and genotype for both mutant mouse lines was observed (two-way ANOVA, *P*_*P30*_ = *0.0359*, F(4,42) = 2.839, *P*_*P70*_ < 0.0001, F(4,54) = 37.99), highlighting the significant differences of *cis*-F-d-proline uptake. At P70, accumulation of *cis*-F-d-proline was significantly higher in the thalamus of *Clcn3*^*td/td*^ mice in comparison to WT, and accumulation in the thalamus of *Clcn3*^*−/−*^ was even higher. Overall, it suggests a potential deficiency of ClC-3-related 2Cl^−^/H^+^ exchange activity in the *Clcn3*^*td/td*^ conditions.

Accumulation of *cis*-F-d-proline in brain areas with neurodegeneration is known to be associated with a prominent reaction of microglia and astrocytes as a sign of neuroinflammation^31,37^. Therefore, to substantiate the *cis*-F-D-proline observations, we performed histopathological stainings in the *Clcn3*^*td/td*^ and *Clcn3*^*−/−*^ brains and quantified the extent of neurodegeneration in the compromised brain areas, such as the thalamus, of these animal models.

Within the VBC of *Clcn3*^*td/td*^ and *Clcn3*^*−/−*^ mice, higher levels of immunoreactive (IR) areas positive for GFAP and CD11b were observed, approximately fivefold higher in *Clcn3* models compared to the WT (Fig. 2A, C and Table 2), suggesting activation of neuroinflammatory processes in the thalamus of both mutant mouse models (WT, *n* = 10*, Clcn3*^*td/td*^ and *Clcn3*^*−/−*^* n* = *6* two-way RM ANOVA, *P* < 0.001, *F*(2,19) = 51.89). Comparison of the models with each other revealed statistically significantly higher levels of CD11b-positive but not GFAP-positive cells in *Clcn3*^*−/−*^ in this brain region (Fig. 2C). Despite the significant loss of neuronal nuclei and activation of astrocytes and microglia in the thalamus in both mouse lines, the adult *Clcn3*^*td/td*^ hippocampus remained unaltered (Suppl. Fig. 1C,G). No decrease in size, no deformation and no degeneration was observed in the hippocampi of adult *Clcn3*^*td/td*^ mice. In agreement with Stobrawa, et al.^20^, at P70, the hippocampus of *Clcn3*^*−/−*^ was absent in contrast to that of *Clcn3*^*td/td*^ mice (Suppl. Fig. 1C,E). *Clcn3*^*−/−*^ and *Clcn3*^*td/td*^ striatum did not show obvious alterations in the neuronal distribution or number of reactive astrocytes compared to the WT striatum (Suppl. Fig. 1B,D,F,G). Moreover, higher levels of IR for GFAP were detected in the brainstem but not in the motor cortex of *Clcn3*^*td/td*^ mice (Suppl. Fig. 2). These observations support the notion that the radiotracer *cis*-F-d-proline preferentially accumulates in brain areas with neurodegenerative processes and identified neuronal atrophy accompanied by gliosis in the *Clcn3*^*td/td*^ thalamus.

### Differential ClC-4 expression levels between hippocampus and thalamus in wild-type mice

**Fig. 3:**
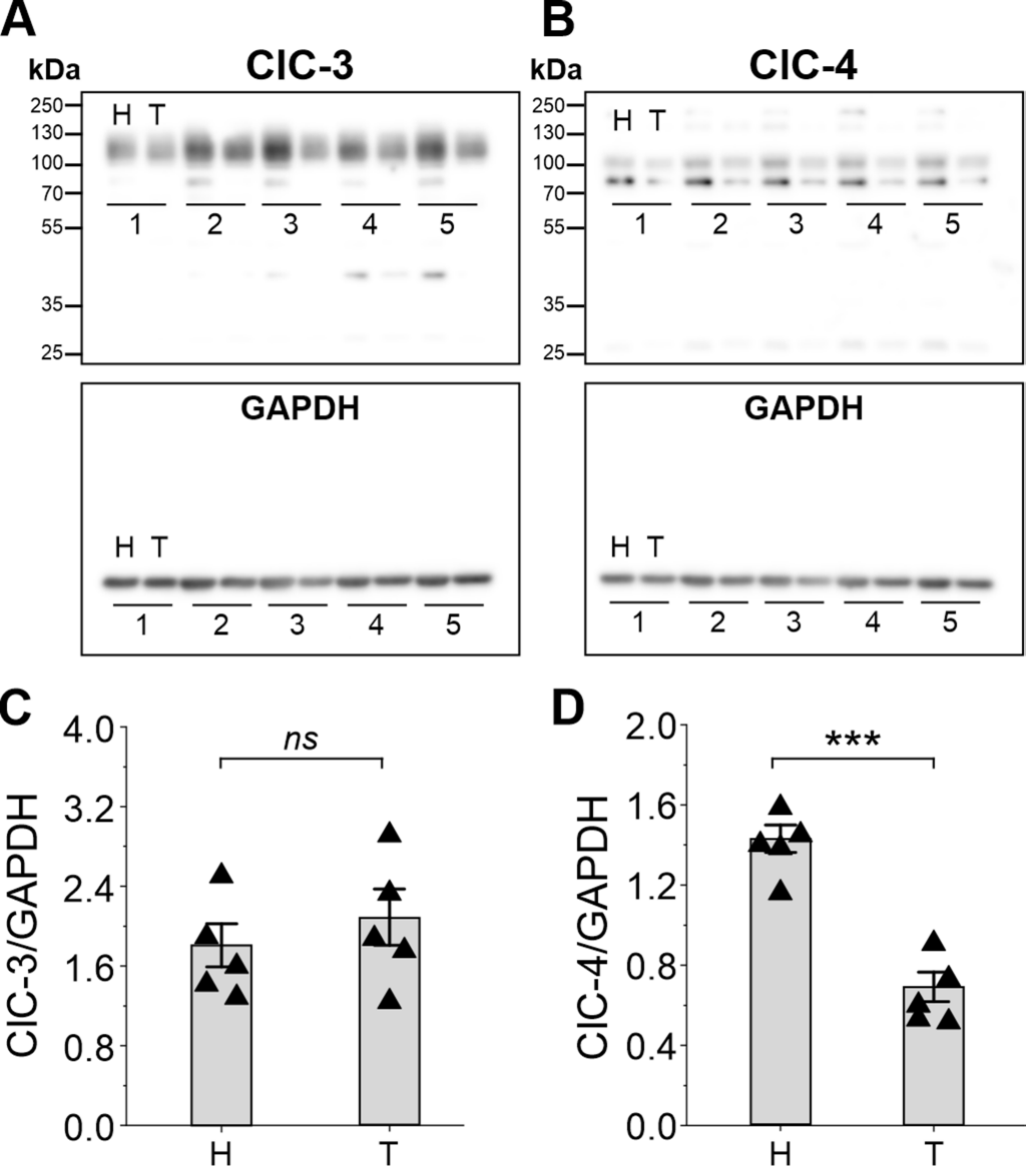
Brain region-specific expression levels of ClC-3 and ClC-4. To test for relative expression levels, immunoblots of protein preparations from the hippocampus (H) and thalamus (T) of five WT (black up triangles) mice were probed with antibodies against ClC-3 (**A**) and ClC-4 (**C**) as well as GAPDH for normalisation. Quantification revealed no difference in ClC-3 expression between hippocampus and thalamus (**B**), whereas ClC-4 levels were significantly lower in the thalamus than in hippocampus (**D**). *n* = 5 mice. Error bars represent the standard error of the mean. Individual values are shown as triangles. ***P < 0.001 for the Wilcoxon-Mann–Whitney rank test.

The above findings imply that the primary reason for degeneration in the thalamus of *Clcn3*^*td/td*^ and *Clcn3*^*−/−*^ mice, but not in the hippocampus of *Clcn3*^*td/td*^ animals, is the absence of 2Cl^−^/H^+^ exchange activity in the thalamus of both animal models. Given that ClC-3^*td/td*^ conserves its ability to heterodimerize with its paralog ClC-4^21^, the lack of hippocampal degeneration in the *Clcn3*^*td/td*^ mouse brains could be attributed to the presence of ClC-4 in this brain region, which ensures the maintenance of endosomal 2Cl^−^/H^+^ exchange activity, preventing degeneration^21^. In contrast, low levels of ClC-4 expression in the thalamus, a scenario that likely leads to the formation of homomeric ClC-3^*td/td*^/ClC-3^*td/td*^ dimers, might result in the loss of 2Cl^−^/H^+^ transport function and subsequent thalamic degeneration in *Clcn3*^*td/td*^ mice. Consistent with this hypothesis, qRT-PCR experiments revealed low levels of ClC-4 mRNA transcripts in the WT thalamus, while ClC-3 mRNA levels were substantially higher (mRNA levels relative to 18S in WT for ClC-4; 0.00254 ± 5.31577E-4, *n* = 4 mice *vs* ClC-3; 0.37211 ± 0.03661, *n* = 3 mice). As the quality of the cDNAs and primer binding efficiencies may vary and affect the calculation of fold changes on gene expression, we conducted immunoblotting of thalamic lysates using anti-ClC-3 and ClC-4 antibodies and compared the relative ClC-3 and ClC-4 protein levels with those in the hippocampus (Fig. 3). Whereas WT hippocampus and thalamus showed comparable proteins levels for ClC-3 (Fig. 3A and 3B), the levels of ClC-4 were significantly lower in the thalamus than in the hippocampus (Fig. 3C and 3D). These observations suggest a differential expression pattern for ClC-4 between the thalamus and hippocampus. They are consistent with a scenario where a significant proportion of ClC-3 forms homodimeric ClC-3/ClC-3 complexes in the WT thalamus, while ClC-3/ClC-4 heterodimers are predominant in the hippocampus.

### Homeostatic regulation of electrophysiological properties in thalamic neurons requires transport-competent 2Cl^−^/H^+^ exchangers

**Fig. 4:**
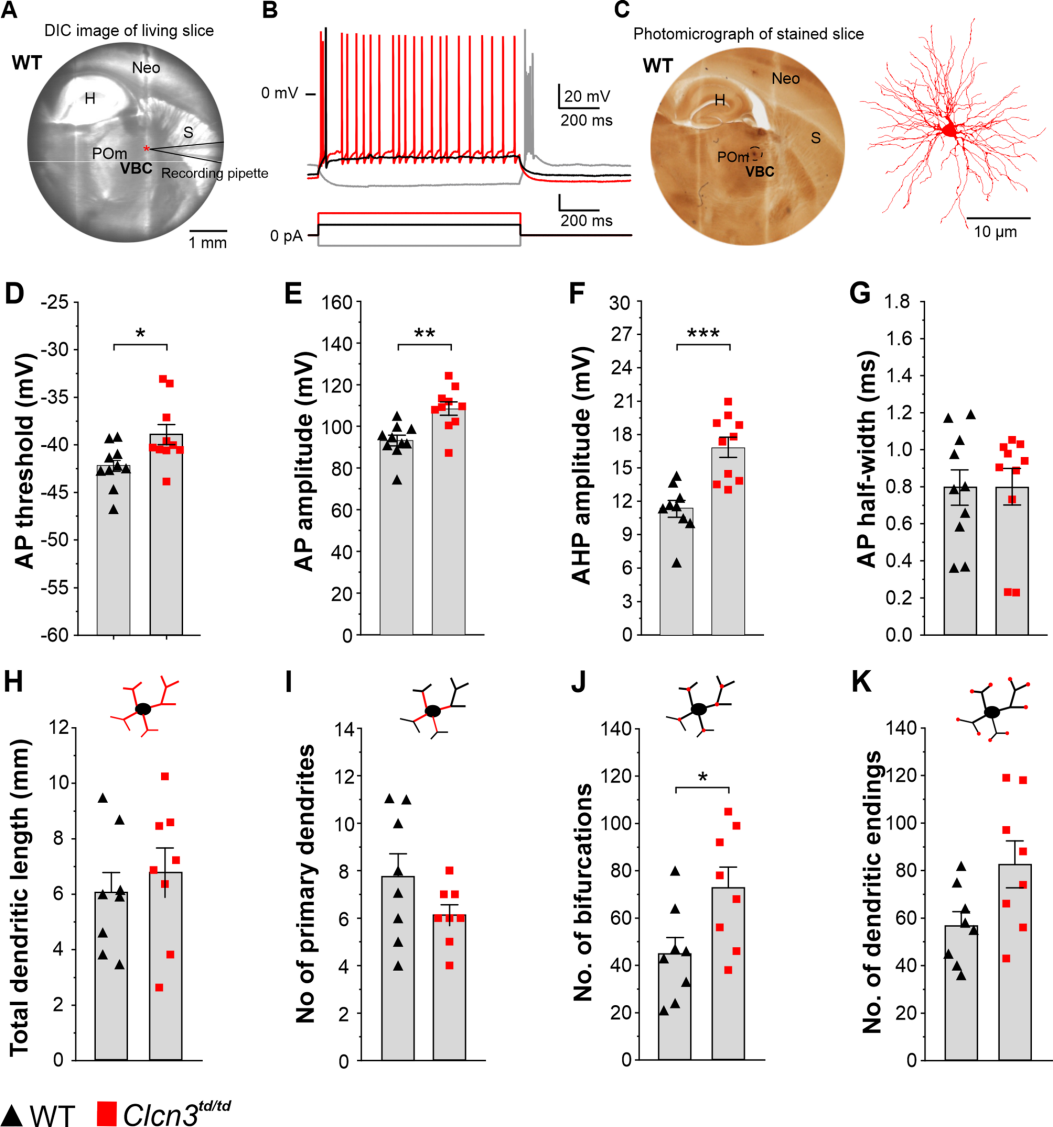
Electrophysiological and morphological properties of thalamic neurons from WT and *Clcn3*^*td/td*^ mice. (**A**) Differential interference contrast (DIC) image (left) of a living thalamocortical slice during whole-cell patch-clamp recordings from a thalamic neuron (asterisk). Hippocampus (H), striatum (S) and neocortex (Neo) are marked for orientation. (**B**) Representative patch-clamp electrophysiological trace from a WT thalamic neuron, illustrating the firing behaviour in response to varying levels of current injection. (**C**) the corresponding photomicrographs of the neuron shown in (A) after fixation and staining. The posterior medial nucleus (POm) is located on the left side of ventrobasal complex (VBC) (lighter area). The stained thalamic neuron outlined with a dotted circle. A somatodendritic reconstruction of the patched thalamic neuron from (**A**) is shown on the right. Electrophysiological parameters such as AP threshold (**D**), AP amplitude (**E**), AHP amplitude (**F**) were significantly different in the P60 *Clcn3*^*td/td*^ neurons when compared to the WT. In contrast, AP half-width (**G**) and most of the neuron’s morphological parameters (**H**,**I**,**K**) did not differ between phenotypes except for number of bifurcations nodes (**J**). H, hippocampus. Neo, Neocortex. WT, *n* = 10 and *Clcn3*^*td/td*^, *n* = 10 thalamic neurons. Error bars represent the standard error of the mean. Individual values are overlaid as black up triangles for WT and red squares for *Clcn3*^*td/td*^. *P < 0.05, **P < 0.01, ***P < 0.001 for the Wilcoxon-Mann–Whitney rank test.

In the majority of neurodegenerative disorders, neuronal death is functionally linked to alterations in the electrical properties of neurons^38^. To investigate this, we examined neurons located at the edge of the neurodegenerative area in the thalamus (Fig. 4). Using patch-clamp recordings with acute brain slices prepared from P60 *Clcn3*^*td/td*^ and WT mice, we evaluated the electrophysiological and morphological properties of thalamic neurons within the VBC. Neurons were patched with biocytin-containing pipettes, histologically processed after the recordings and subsequently morphologically reconstructed (Fig. 4A,B). ClC-3 transport-deficient *Clcn3*^*td/td*^ thalamic neurons exhibited significant changes in their electrophysiological properties. Active properties such as action potential (AP) threshold, amplitude, and the amplitude of the afterhyperpolarization (AHP) were significantly altered in the *Clcn3*^*td/td*^ thalamic neurons compared to WT neurons (AP threshold WT -42.2 ± 2.3 mV vs *Clcn3*^*td/td*^ -38.9 ± 3.4 mV, absolute/relative increase 3.3 mV/7.8%; AP amplitude WT 93.1 ± 8.2 mV vs *Clcn3*^*td/td*^ 108.5 ± 10.3 mV, absolute/relative increase 15.4 mV/16.5%; AHP amplitude WT 12.3 ± 3.8 mV vs *Clcn3*^*td/td*^ 16.8 ± 2.9 mV, absolute/relative increase 4.5 mV/36.6%; AP half-width WT 0.795 ± 0.303 ms vs *Clcn3*^*td/td*^ 0.799 ± 0.313 ms; absolute/relative increase 0.004 ms/5.0%). Figure 4C–E and Suppl. Table 1). The AP threshold was shifted to more depolarizing potentials, and the amplitude of both AP and AHP was higher in the mutant than in the WT (Fig. 4D and 4E). In contrast, no differences were observed in the passive electrical properties of the thalamic neurons (Suppl. Table 1). Resting membrane potential, membrane input resistance, and time constant τ_m_ in *Clcn3*^*td/td*^ did not differ from WT (Suppl. Table 1). Both *Clcn3*^*td/td*^ and WT thalamic neurons retained the ability to generate rebound bursting near the end of negative current injection, attributed to the activation of low-threshold Ca^2+^ currents^39^. Changes in action potential properties suggest that the 2Cl^−^/H^+^ exchange mediated by ClC-3 is essential for maintaining the proper electrophysiological features of thalamic neurons.

Dendrites represent the integrative units of neurons, and alterations in their morphological properties may be causally related to aberrant electrophysiological characteristics^40^. Therefore, we reconstructed the 3D morphology of the recorded neurons and performed a detailed morphometric analysis of *Clcn3*^*td/td*^ and WT thalamic neurons. The total dendritic length, the number of primary dendrites, their branching pattern, and the number of terminal endings were examined. Except for the number of bifurcation nodes, no major differences between *Clcn3*^*td/td*^ and WT mice were identified (Fig. 4G–J and Suppl. Fig. 3). The higher number of bifurcation nodes for *Clcn3*^*td/td*^ compared to WT mice indicates an increased complexity in the branching pattern of *Clcn3*^*td/td*^ neurons. These observations exclude the possibility that alterations in the electrophysiological properties of *Clcn3*^*td/td*^ thalamic neurons are due to changes in dendritic morphology. Conversely, they emphasize the crucial role of 2Cl^*−*^/H^+^ exchange activity in ensuring optimal neuronal function.

### Behavioural phenotype of ***Clcn3***^***td/td***^ mice

**Fig. 5:**
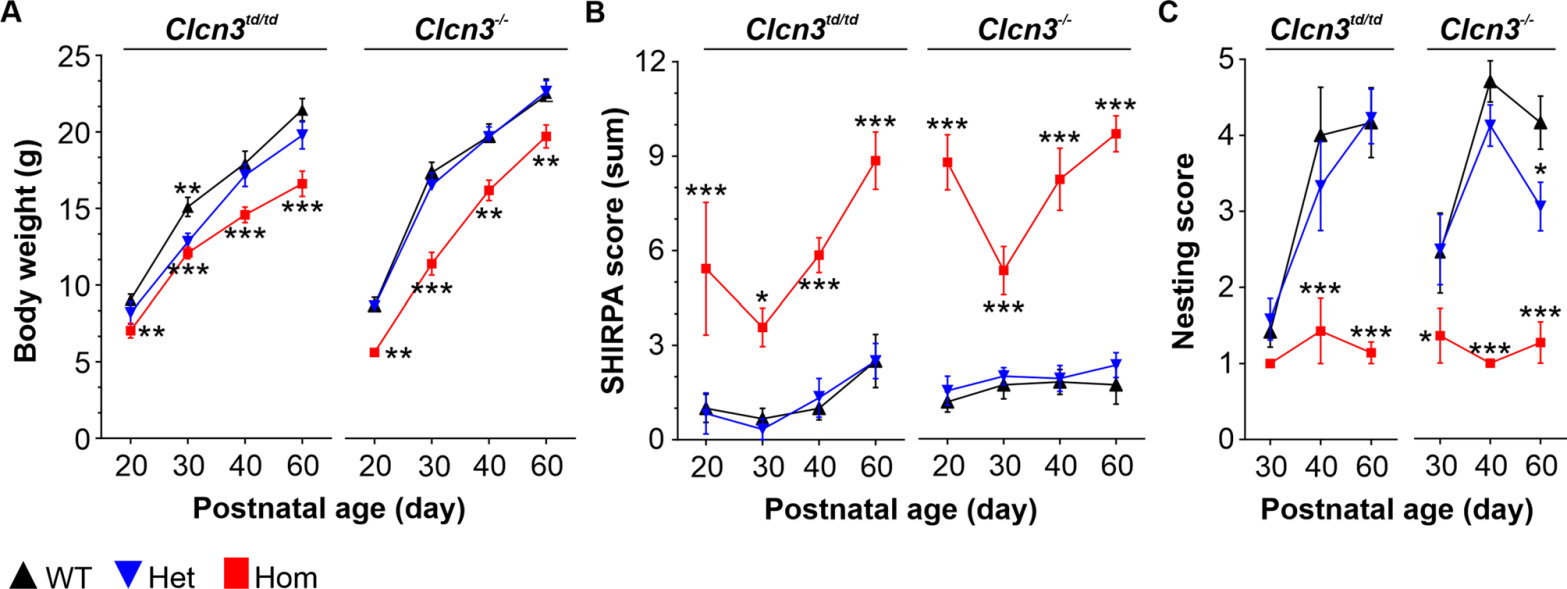
Phenotypic and behavioural characterization of *Clcn3*^*td/td*^ and *Clcn3*^*−/−*^ mice revealed similarities in their impaired behaviour. (**A**) Homozygous (red squares) *Clcn3*^*td/td*^ and *Clcn3*^*−/−*^ mice showed significantly lower body weights during postnatal development compared to their WT (black up triangles) and heterozygous (blue down triangles) littermates. WT *knock-in*, *n* = 12; *Clcn3*^+*/td*^, *n* = 6 and *Clcn3*^*td/td*^, *n* = 12; WT *knock-out*, *n* = 11; *Clcn3*^+*/−*^, *n* = 13 and *Clcn3*^*−/−*^, *n* = 12. (**B**) Higher SHIRPA scores in *Clcn3*^*td/td*^ and *Clcn3*^*−/−*^ mice across a longitudinal experimental setup spanning 40 days indicate a significant lower overall sensory perception and motor coordination. (**C**) Nesting performance remained significantly low for *Clcn3*^*td/td*^ and *Clcn3*^*−/−*^ mice, while *Clcn3*^+*/td*^ and *Clcn3*^+*/−*^ and WT littermates began to build nests and significantly improved their nesting performance during maturation. WT *knock-in*, *n* = 6; *Clcn3*^+*/td*^, *n* = 6 and *Clcn3*^*td/td*^_*,*_* n* = 7; WT *knock-out*, *n* = 11; *Clcn3*^+*/−*^* n* = 14 and *Clcn3*^*−/−*^, *n* = 11. Data are presented as mean ± SEM. Statistical calculations were conducted by Repeated Measures Parametric ANOVA with Fisher *post-hoc* test. Significant data for groups compared to WT are marked by asterisks. *P ≤ 0.05, **P ≤ 0.01, ***P ≤ 0.001.

To investigate the impact of the thalamic degeneration in *Clcn3*^*td/td*^ mice on animal behaviour, we aimed at a longitudinal characterization of *Clcn3*^*td/td*^ and *Clcn3*^*−/−*^ mice at different developmental stages, ranging from P20 to P60, in comparison to their heterozygous and WT littermates. Overall, *Clcn3*^*td/td*^ mice were viable and fertile and showed no obvious differences in anatomy and behaviour in their home cage. On average, *Clcn3*^*td/td*^ mice were slightly smaller than WT or *Clcn3*^+*/td*^, with reduced body weight at all ages tested (Fig. 5A). Body weight generally increased with age (two-way RM ANOVA, *Clcn3*^*td/td*^* P* < 0.0001, *F*(3,72) = 48.37 and *Clcn3*^*−/−*^* P* < 0.0001, *F*(3,90) = 93.98). Significant differences in body weight development were observed in mutant mice (two-way RM ANOVA, interaction genotype per age, *Clcn3*^*td/td*^* P* = 0.0064, *F*(6,72) = 3.29 and *ClCn3*^*−/−*^* P* = 0.0067, *F*(6,90) = 3.21). Despite these differences, *Clcn3*^*td/td*^ mice reached a normal lifespan without premature death, in contrast to 70% of the *Clcn3*^*−/−*^ mice^20^.

**Fig. 6:**
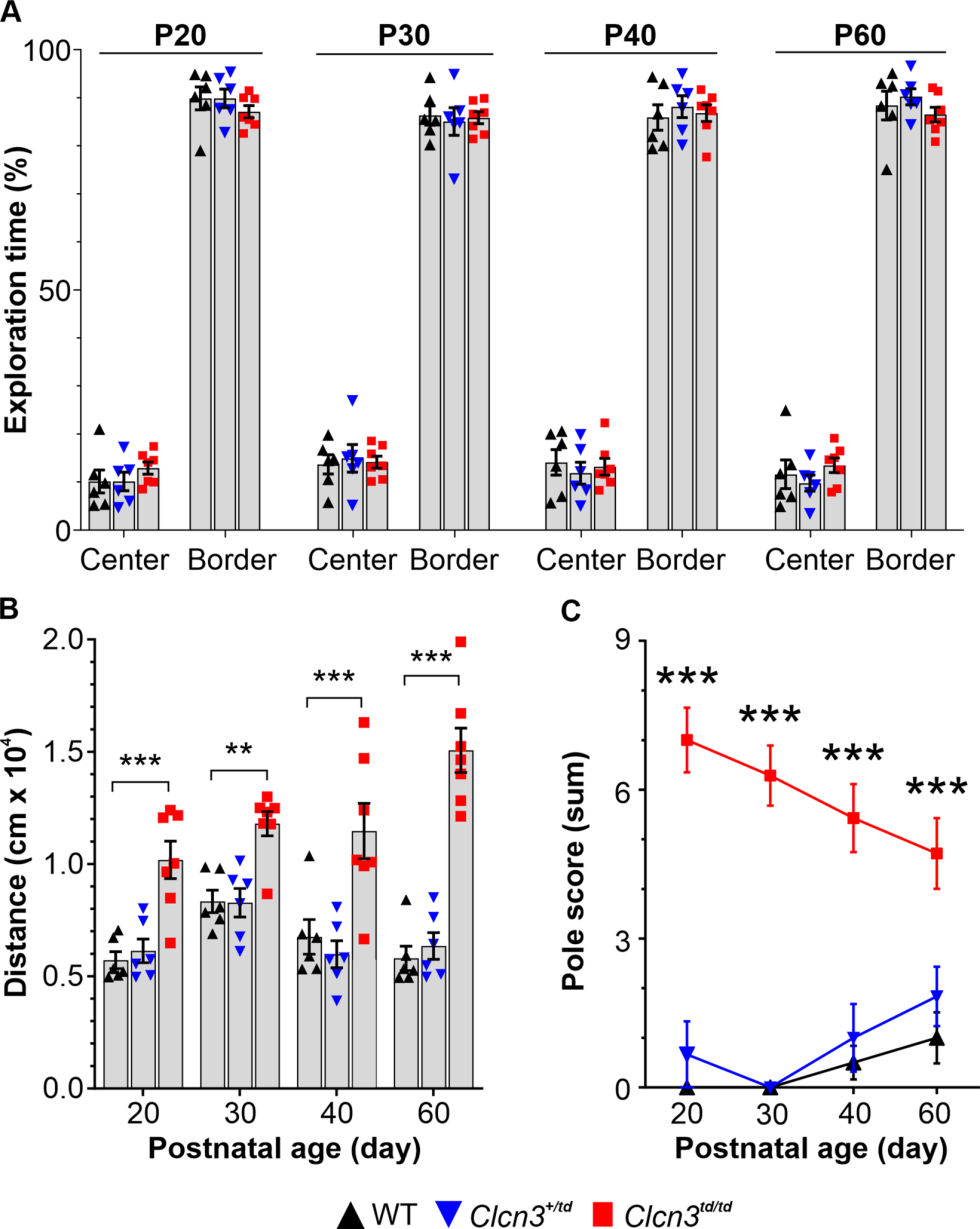
*Clcn3*^*td/td*^ mice exhibited hyperactivity and dysfunctions in their motor coordination in the open field and pole test. (**A**) *Clcn3*^*td/td*^ (red squares) mice exhibited similar exploration times in the defined centre or border zones of the open field arena compared to *Clcn3*^+*/td*^ (blue down triangles) and WT (black up triangles) littermates. However, they demonstrated significantly higher total movement distance, indicating hyperactivity throughout postnatal development (**B**). The moved distance was progressively higher in *Clcn3*^*td/td*^ mice during postnatal development, further highlighting their hyperactive behaviour. (**C**) Evaluation of the pole test, assessing basal ganglia-associated movement dysfunctions, revealed significantly impaired performance in *Clcn3*^*td/td*^ mice, with a trend towards improvement during maturation. Data are presented as mean ± SEM. Statistical calculations were conducted by Repeated Measures Parametric ANOVA with Fisher post hoc test WT vs. *Clcn3*^*td/td*^*.* WT, *n* = 6; *Clcn3*^+*/td*^, *n* = 6; *Clcn3*^*td/td*^, *n* = 7. Significant data are marked by asterisks. **P ≤ 0.01, ***P ≤ 0.001.

Primary screening with the SHIRPA test battery revealed overall lower levels of sensory perception and motoric abilities (cornea reflex, alertness, startle and touch response, hanging behaviour) resulting in significantly higher SHIRPA score values for *Clcn3*^*td/td*^ when compared to their littermate controls and *Clcn3*^*−/−*^ (*Clcn3*^*td/td*^; *P* = 0.0028, F(2,16) = 8.69 and *Clcn3*^*−/−*^; *P* < 0.0001, F(2,34) = 52.72, two-way RM ANOVA, Fig. 5B and Suppl. Table 2). The drop in SHIRPA scores for both mouse lines at P30 likely indicated age-related improvements of the mice during maturation, followed by progressive dysfunction during aging. Nest-building capabilities were assessed as indicators of well-being and complex motor functions (Fig. 5C and Suppl. Table 2). At P30, regardless of their genotype, all mice demonstrated relatively low nest-building abilities. Whereas *Clcn3*^+*/td*^*, Clcn3*^+*/−*^, and WT mice improved nesting abilities with age, reaching a nesting score of ~4 at P60, *Clcn3*^*td/td*^ and *Clcn3*^*−/−*^ mice showed a significantly impaired nesting performance (*Clcn3*^*td/td*^; *P* < 0.001, *F*(2,32) = 16.06 and *Clcn3*^*−/−*^; *P* = 0.0005, *F*(2,66) = 8.45, two-way RM ANOVA, Fig. 5C and Suppl. Table 2). *Clcn3*^+*/−*^ mice showed a moderate, yet significant decline in nesting performance at P60 compared to their WT littermates. In contrast, *Clcn3*^*td/td*^ and *Clcn3*^*−/−*^ mice were completely incapable of learning and building complete nests (Fig. 5C and Suppl. Table 2). To assess locomotor, explorative, and anxiety-related behaviour, we performed an open field test with the *Clcn3*^*td/td*^ mice (Fig. 6). There was no discernible difference in the explorative behaviours among WT, *Clcn3*^+*/td*^, and *Clcn3*^*td/td*^ mice, with all mice spending more than 80% of the evaluated time at the border zones and less than 20% at the centre (Fig. 6A). Although these observations indicate no abnormalities in the explorative behaviour, *Clcn3*^*td/td*^ mice showed significantly higher overall locomotion. They covered almost twice the distance travelled by their littermates (two-way RM ANOVA, *P* = 0.0008, *F*(2,16) = 11.37, Fig. 6B), suggesting hyperactivity. Additionally, a significantly altered locomotion over time was observed (two-way RM ANOVA, interaction genotype per age, *P* = 0.0012 *F*(6,48) = 4.43).

To analyse basal ganglia-associated movement dysfunction in *Clcn3*^*td/td*^ mice, the vertical pole test was performed (Fig. 6C). Generally, pole performance was significantly impaired in *Clcn3*^*td/td*^ mice across all ages compared to their littermates (two-way RM ANOVA, *P* < 0.0001, *F*(2,16) = 50.95) as highlighted by high pole score values (Fig. 6C and Suppl. Table 2). In addition, statistical analyses showed a significant alteration in pole performance over time, with lower pole scores observed during development (two-way RM ANOVA, interaction genotype x age, *P* = 0.0081, *F*(6,48) = 3.33). In contrast to WT and *Clcn3*^+*/td*^, pole performance in *Clcn3*^*td/td*^ declined with age. These findings suggest that ablation of the 2Cl^−^/H^+^ exchange function of ClC-3 leads to neuronal dysfunction and subsequent thalamic degeneration, ultimately resulting in behavioural deficits.

## Discussion

Using the PET-radiotracer *cis*-F-d-proline technology in combination with the transport-deficient *Clcn3*^*td/td*^ knock-in and *Clcn3*^*−/−*^ mouse models and electrophysiological recordings, we have uncovered a brain region in which the functionality and viability of neuronal cells tightly depend on the 2Cl^−^/H^+^ exchange function of ClC-3 homodimers. Notably, we found a substantial accumulation of the radiotracer *cis*-F-d-proline in the thalamus of *Clcn3*^*−/−*^ and *Clcn3*^*td/td*^ mouse models (Fig. 1), which correlates with a significant reduction in the neuronal population and the activation of neuroinflammatory processes (Fig. 2). *Clcn3*^*td/td*^ thalamic neurons exhibited aberrant electrical properties (Fig. 4). In multiple behavioural tests, we primarily observed a motor phenotype of *Clcn3*^*td/td*^ mice, suggesting a link between thalamic degeneration, altered neuronal excitability, and behavioural dysfunctions in this mouse model (Fig. 4 and 5).

ClC-3 and ClC-4 are co-expressed in the hippocampus, and the integrity of this brain region depends on the presence of functional ClC-3^18–20^ as well as ClC-3/ClC4 heterodimers^21,30^. Disentangling deficits in ion transport mediated by ClC-3 homodimers versus ClC-3/ClC-4 heterodimers in the brain is challenging. Therefore, to investigate which brain regions, in addition to the hippocampus, are functionally dependent on the presence of ClC-3 homodimers, we used a transport-deficient *Clcn3*^*td/td*^ mouse and employed a highly sensitive radiotracer^31,32^ to detect neurodegenerative processes in the brain of this animal model. For comparison, *Clcn3*^*−/−*^ and WT mice were used. We found neurodegeneration in the thalamus of both mutant mouse models, but not in the hippocampus of *Clcn3*^*td/td*^ mice. Neurodegeneration in the thalamus of *Clcn3*^*td/td*^ mice was unexpected, since *Clcn3*^*td/td*^ carries a point mutation (p.E281Q) that abolishes the anion transport function of ClC-3 without affecting its capability to heterodimerize with ClC-4^21^. Therefore, the absence of neurodegeneration in the hippocampus of *Clcn3*^*td/td*^ mice could be primarily ascribed to the presence of functional ClC-4 as heterodimeric ClC-3/ClC-4 complexes^21,30^. We hypothesized that the absence of ion transport by ClC-3/ClC-4 heterodimers in the thalamus results in neuronal dysfunction and subsequently degeneration within this brain region. Although, at P30, neurodegeneration and inflammation were comparable between *Clcn3*^*−/−*^ and *Clcn3*^*td/td*^ mice, at P70, the accumulation of *cis*-F-d-proline and levels of CD11b-positive cells increased further in the thalamus of *Clcn3*^*−/−*^ mice only. However, the number of neurons in this brain region was equally low at P70, arguing for a comparably severe neurodegeneration triggered by the loss of the transport function and the complete absence of ClC-3, respectively.

The endosomal presence of ClC-4 depends on the protein levels of ClC-3 and ClC-4^8,30^. Therefore, higher ClC-3 levels relative to ClC-4 in a given brain region predict a preferential assembly of homodimeric ClC-3 complexes. We demonstrated that in WT thalamus and hippocampus, ClC-3 protein levels are similar, while ClC-4 levels are lower in the thalamus compared to the hippocampus (Fig. 3). This scenario is consistent with our hypothesis and suggests an excess of ClC-3 resulting in the formation of ClC-3 homodimers and less ClC-3/ClC-4 heterodimers in the WT thalamus. Therefore, it is conceivable to assume a predominant association of ClC-3/ClC-4 in the hippocampus and of homodimeric ClC-3/ClC-3 in the thalamus. Indeed, the *Clcn3*^*td/td*^ thalamus recapitulated the phenotype observed in the hippocampus of the double mutant mice *Clcn3*^*td/td*^/*Clcn4*^*−/−*^ and *Clcn3*^*unc/unc*^/*Clcn4*^*−/−*^, wherein the absence of 2Cl^−^/H^+^ endosomal function leads to loss of neurons^21,30^. Thus, we concluded that the viability of thalamic cells critically depends on endosomal 2Cl^−^/H^+^ exchange provided by ClC-3 homodimers.

Neuronal death is the central pathogenic process in all neurodegenerative disorders. Gaining insight into the events preceding neuronal loss, such as alterations in the electrophysiological properties of neurons, is crucial for understanding the underlying molecular mechanisms of the disease^41^. *Ex vivo* electrophysiological examination of *Clcn3*^*td/td*^ neurons in the vicinity of the degenerating area in the thalamus showed changes in the action potential waveform. We found a depolarizing shift in the spike threshold, larger AP amplitudes, and higher AHP values. The AP threshold is a readout of cell excitability and depends on the properties and density of Na^+^ channels. However, K^+^ channels can play an adaptive role. A shift to more depolarizing potentials in the excitability threshold indicates that *Clcn3*^*td/td*^ neurons are less excitable than WT. The activation kinetics and density of Na^+^ channels drive the rising phase of the AP. In this context, higher AP amplitudes may reflect a larger density of Na^+^ channels in the *Clcn3*^*td/td*^ condition. However, less excitable *Clcn3*^*td/td*^ cells with more Na^+^ channels at the plasma membrane are counterintuitive. Thalamic neurons express Na_v_1.1, Na_v_1.2, Na_v_1.3, and Na_v_1.6 sodium channels^42^, and all of them can modulate the action potential threshold^43^. Among these, Nav1.3 exhibits the highest threshold^43^. Thus, it is tempting to speculate that a defect in Na_v_1.3 expression or trafficking contributes to the observed changes in the action potential waveform in *Clcn3*^*td/td*^ neurons. Given that direct evidence linking ClC-3 transport deficiency to Nav1.3-specific dysregulation is currently lacking, further investigation is required to determine whether ClC-3 regulates expression, localization, or functional modulation of Nav1.3 or other Nav isoforms. AHP is typically delineated by the activation of large-conductance calcium-activated potassium channels (BK), K_v_7 (KCNQ), small-conductance calcium-activated potassium channels (SK) and hyperpolarization-activated cyclic nucleotide-gated (HCN) channels, all of which can be found in the thalamus^44–46^. The mechanisms controlling the magnitude of AHP are important determinants of neuronal excitability, as they serve to regulate the AP thresholds^47^. We have recently demonstrated that Cl⁻/H⁺ exchangers are critical regulators of neuronal excitability in hippocampal pyramidal neurons^48^. Specifically, we showed that ClC-3 modulates action potential threshold and the amplitude of the AHP by regulating the density of Kv7/KCNQ potassium channels^48^. In the present study, the voltage sag, a hallmark feature associated with HCN channel activity, was not different between WT and *Clcn3*^*td/td*^ neurons (WT 21.1% ± 5.5 vs *Clcn3*^*td/td*^ 14.5% ± 6.3, *P* = 0.10), suggesting that HCN channels are unlikely to contribute to the increased AHP. This evidence strongly suggests that the increased AHP amplitude observed in *Clcn3*^*td/td*^ neurons is more likely due to an increase in Kv7/KCNQ channel density rather than BK, SK, HCN channels or a homeostatic compensatory mechanism such as altered ion channel expression.

The enhanced AHP and a depolarizing shift in the action potential threshold in *Clcn3*^*td/td*^ are consistent with the notion that the 2Cl^−^/H^+^ exchange activity of ClC-3 is required for maintaining the electrogenic balance in neurons^21^. Given the existence of two major ClC-3 isoforms in the CNS, with ClC-3b found in lysosomes and ClC-3c enriched in recycling endosomes^9^, both lysosomal and recycling endosomal defects could be responsible for the mutant phenotype in *Clcn3*^*td/td*^. However, while neurons also express ClC-6 and ClC-7, the predominant lysosomal Cl^*−*^/H^+^ exchangers^10^, only ClC-3c is present in recycling endosomes^9^. This compartment-specific distribution suggests that, in *Clcn3*-deficient or *Clcn3* transport-deficient neurons, recycling endosomes are more severely affected than lysosomes. While disruptions in endosomal pH, in particular within lysosomes, can impair protein sorting and degradation, we propose that recycling endosomes may be more affected in the *Clcn3*^*td/td*^ model, as they lack compensatory Cl^*−*^/H^+^ exchangers such as ClC-6 and ClC-7^9,10,17^. *Clcn7*^*−/−*^ mice, which lack one of the major lysosomal Cl^*−*^/H^+^ exchangers, exhibit a more severe neurological phenotype than *Clcn3*^*−/−*^ mice^17,20^, reinforcing the view of compartment-specific vulnerability among CLC transporters. Our data, from two independent neuronal types^48,49^, support a model in which ClC-3-dependent alterations in intrinsic excitability contribute significantly to neuronal function, vulnerability and degeneration. While the contribution of lysosomes’ pH disruption should not be overlooked, excitability changes, particularly through Kv7/KCNQ channel regulation, represent a crucial and underappreciated mechanism underlying ClC-3 function in neuronal survival and plasticity. Altogether, this evidence suggests that a defect in the mechanisms regulating the trafficking of important ions such as Na^+^ and K^+^ is the most likely cause of neuronal electrical failure and, thus, thalamic neurodegeneration in the *ClCn3*^*td/td*^ mouse model. Consistent with this, the observed alteration in the number of dendritic bifurcations may reflect a less differentiated neuronal morphology, potentially resulting from dendritic retraction associated with the early stages of neurodegeneration. Due to its central role as a relay station of many afferent and efferent neuronal connections, the thalamus is prone to neurodegeneration, as shown for multiple sclerosis and other disease conditions^50,51^.

The behavioural characterization of the *Clcn3*^*td/td*^ mice revealed deficits primarily associated with motor function, including locomotion, motor strength and motor coordination. These deficits were evident across different ages and revealed by multiple assessments such as the SHIRPA test battery, open field test, pole test and nest building abilities (Fig. 5 and Fig. 6), resembling the motor deficit observed in the *Clcn3*^*−/−*^ mice. *Clcn3*^*td/td*^ and *Clcn3*^*−/−*^ mice exhibited reduced body weight compared to their WT littermates and performed very similarly in the SHIRPA test battery and the nest building test. Furthermore, *Clcn3*^*td/td*^ mice displayed clear signs of hyperactivity in the open field test, consistent with previous observations in *Clcn3*^*−/−*^ mice^19,20^. Impairments in motor coordination were evident from the pole test, although improved performance with repeated training suggested intact motor learning, a characteristic also noted in *Clcn3*^*−/−*^ mice^20^. However, for an exact comparison of the motor learning and pole test performance of the two lines, side-by-side comparisons within the same lab would have been necessary. Nevertheless, additional deficits in motor strength and coordination, assessed with both lines by the hanging wire test as part of the SHIRPA test battery, were consistent with findings reported by Yoshikawa, et al.^19^ for their *Clcn3*^*−/−*^ mouse model, and were similarly observed in both *Clcn3*^*td/td*^ and *Clcn3*^*−/−*^ lines. Previously, we reported an additional phenotype in *Clcn3*^*−/−*^ mice, i.e. higher sensitivity towards thermal and chemical noxious stimuli^21^ and similar results were obtained for the *Clcn3*^*td/td*^ line (Suppl. Fig. 4). Given that the somatosensory system responsible for nociception involves nuclei of the VBC in the thalamus^52,53^, these findings suggest a link between the observed pain-sensitivity phenotype and the neurodegeneration identified in the thalamic region of *Clcn3*^*td/td*^ and *Clcn3*^*−/−*^ mice. An additional explanation for the higher pain sensitivity of *Clcn3*^*td/td*^ mice could be the enhanced electrical activity of peripheral nociceptors and inflammatory processes in the spinal cord, as observed in *Clcn3*^*−/−*^ mice^21^. The connectivity of the VBC to visual inputs has been previously documented^54^, suggesting a potential link between thalamic neuron loss and the retinal degeneration observed in *Clcn3*^*−/−*^ and *Clcn3*^*td/td*^ mice, as shown by Sierra-Marquez, et al.^21^. In addition, the VBC is interconnected to the pons in the brainstem^55,56^, where we found higher levels of neuroinflammation. This inflammatory response and dysfunction within the pons may contribute to disturbances in motor function. Indeed, an imbalance between inhibitory and excitatory neurons in the brainstem has been associated with hyperactivity and ADHD-like behaviour^57^. Brain MRI scans of human *CLCN3* mutant carriers revealed several neuroanatomical differences, including misfolded hippocampi, hypoplastic pons and corpus callosum, and lower white matter volume^13,15^. While we did not observe overall changes in white matter or corpus callosum in the brains of *Clcn3*^*−/−*^ or *Clcn3*^*td/td*^ mice, the pathological similarities of our animal models with *CLCN3-*related patients in the pons are striking. However, hypoplasia of the thalamus has not yet been reported in *CLCN3* mutant carriers, and detailed analysis of neuroinflammation in this context remains pending.

In summary, the similar phenotypes observed in *Clcn3*^*td/td*^ and *Clcn3*^*−/−*^ mice regarding motor-related deficits correlate with the neurodegenerative processes identified in the thalamus of both mouse lines. However, while neurodegeneration in the thalamus and hippocampus results from a shared functional defect, distinct molecular mechanisms appear to underlie these processes, with hippocampal neuronal loss being specific to *Clcn3*^*−/−*^ mice. Moreover, the observed phenotype in both mutant *Clcn3* mouse lines reflects clinical symptoms reported in *CLCN3-*related patients^13,15^. The reduced body weight and hyperactivity observed in the mutant mice correspond to documented instances of failure to thrive and hyperactivity/restlessness in certain *CLCN3* mutant patients^15^, while delayed motor development and hypotonia in *CLCN3* patients align with deficits observed in mouse behavioural tests. However, the clinical symptoms of patients harbouring *CLCN4* mutations do not clearly correspond with those of *CLCN3* patients, suggesting that ClC-3 and ClC-4 may have distinct roles in neuronal function. The discrete expression patterns of ClC-3 and ClC-4 in the thalamus and hippocampus suggest that the presence of either homodimeric ClC-3 or heterodimeric ClC-3/ClC-4 assemblies within specific brain tissues may be crucial in the disease aetiology of *CLCN3/4* conditions. This underscores the complexity of the interplay between these transporters and their assembly states in the pathogenesis of *CLCN3/4*-related conditions.

In conclusion, our findings emphasize the critical role of endosomal 2Cl^−^/H^+^-exchange activity within the thalamus. The absence of either ClC-3/ClC-4 heterodimers or functional ClC-3 homodimers leads to neurodegeneration in vulnerable brain regions and associated behavioural deficits. Furthermore, our study highlights the significance of the thalamus in understanding the pathophysiology related to *CLCN3*. We propose that combining the PET-tracer *cis*-F-d-proline, which sensitively detects neurodegeneration, with MRI could serve as a valuable clinical tool for detecting pathological changes and monitoring disease progression in patients affected by the rare *CLCN3-* and *CLCN4-*related condition.

## Materials and methods

### Animals

The *Clcn3*^*−/−*^ knock-out mouse line was kindly provided by Dr. T.J. Jentsch (Leibniz-Forschungsinstitut für Molekulare Pharmakologie). *Clcn3*^*+/td*^ knock-in mice were generated by homologous recombination by Cyagen (Cyagen Biosciences Inc, USA) and maintained in-house as heterozygous by continuous cross-breeding with C57BL/6 as previously described^21^. By intercrossing heterozygous *Clcn3*^+*/td*^ mice of either genotype, WT, heterozygous *Clcn3*^+*/td*^, or homozygous *Clcn3*^*td/td*^ mice were generated. WT and *Clcn3*^*−/−*^ mice were used as controls. Genotypes were identified by a specific PCR-based genotyping protocol using the KAPA Mouse Genotyping Kit (Kapa Biosystems/Roche, KK-7302, Wilmington, MA, United States), according to the manufacturer’s instructions. The following set of primers was used: forward 5ʹ-CACGGGATCACAGTAGTGAAAGG-3ʹ and reverse 5ʹCGCTGCAGTCCATTAAACAGTTTC3ʹ; fragments 332 bp and 250 bp for mutant and WT were expected. Mice were housed under controlled conditions with a 12/12 h light/dark cycle (lights on from 7 a.m. to 7 p.m.), humidity of 55 ± 10%, constant room temperature of 22 °C, and food and water available ad libitum.

### Behavioural test setup

Longitudinal characterization of mixed genders of WT, *Clcn3*^+*/td*^ and *Clcn3*^*td/td*^ and *Clcn3*^*−/−*^ mice at the age (mean ± SD) of postnatal 21 ± 2 (P20), 30 ± 2 (P30), 41 ± 3 (P40), 60 ± 4 (P60) based on the following tests: phenotype assessment using the SHIRPA test battery^58^, motor balance and coordination were further investigated with the nesting^59^ and modified pole test^60^. All mice were weighed and habituated for 30 min in single cages prior to tests (except P20 mice, which stayed in their home cages with siblings). Experiments were performed in a chronological order for each cohort, and experimenters were blinded to genotype. Suppl. Table 3 gives an overview of the mice in the study. 

### SHIRPA phenotype assessment

The primary behavioural screen of the SHIRPA test battery was used to evaluate motorsensory behaviour and reflexes^58^. Mice were individually observed and scored in their home-cage or in the arena (B19.5 cm × H55 cm × W33 cm) in the subtests: restlessness, apathy, grooming, stereotyped behaviour, convulsions, abnormal body carriage, alertness, abnormal gait, clasping, hanging wire, startle response, touch response, pinna reflex, cornea reflex, whisker touch response, pain response. Scoring was defined from 0 (similar to WT) to 3 (extremely changed) and summed up as SHIRPA score of each mouse.

### Pole test

Basal ganglia-associated movement dysfunctions were assessed with the modified pole test as previously described^60^. Briefly, mice were placed with their head downwards on top of a vertical pole (50 cm height) to rate their movement from 0 (running continuously) to 3 (not able to move, falling down). The procedure was repeated three times, and summed scores were used for analyses.

### Nesting test

The nesting test was performed to analyse rodents’ natural behaviour in constructing nests with provided materials, aiming to protect their litters and preserve heat^59^. For instance, lesions in hippocampal regions could result in the loss of species-typical behaviour. Thus, the mouse’s nesting competence can provide insights into both its motor coordination and cognitive performance^61^. Mice were housed separately overnight, with food and water ad libitum. A cotton “Nestlet” was offered for environmental enrichment. After 16 h, nests and condition of residues from Nestlets were evaluated by two independent experimenters with a modified 5-point-scoring system by Robert M.J. Deacon: 1 > 90% undamaged; 2, 50 to 90% cotton shredded in cage; 3, < 50% intact, no obvious nest; 4, 100% shredded, flat nest without walls; 5, 100% shredded, perfect surrounding nest.

### Open field test

Anxiety-related, explorative behaviour and locomotion of *Clcn3*^*td/td*^ mice were analysed in the open field test. Therefore, mice were placed in the right corner of a square-shaped arena (L44 cm × W44 cm × H40 cm). Lighting was maintained at 400 to 500 lx during experiments. Mice could move freely for 20 min in the arena, which was virtually divided into a border (11 cm from the wall) and a centre zone (22 cm × 22 cm middle part). Video tracking software EthoVision XT (Noldus Information Technologies Wageningen, Netherlands) was used to evaluate the distance moved in the zones (cm), the time spent in the arena zones (s) and velocity (cm s^−1^).

### *Ex vivo* autoradiography

All animals in this study were anaesthetised with 5% isoflurane supplied at a flow rate of 2 l/min using an anaesthetic vaporiser (Eickemeyer) and subsequently euthanised by decapitation. The tracer *cis*-4-[^18^F]-d-proline (*cis*-F-d-proline) was used to assess potential neurodegeneration and was produced as previously described^62,63^. After anaesthetising, mice (WT, *Clcn3*^*td/td*^ and *Clcn3*^*−/−*^ mice at the age of (mean ± SD) 27 ± 2 (P30) and 67 ± 6 (P70) days) were injected with *cis*-F-d-proline into the tail vein. After 2 h of tail injection, mice were decapitated and used for *ex vivo* autoradiography. The activity of the tracer was set to 15 MBq per 150 µL injection volume. Brains were dissected and snap-frozen in liquid isopentane (− 50 °C). Frozen 20 µm sections of the right brain hemisphere were sliced with a cryostat (Leica CS305 S, Leica Biosystems Nussloch GmbH, Germany). Slices were collected beginning at 3.44 mm to 1.42 mm lateral to Bregma, corresponding to the standard sagittal anatomic mouse brain atlas^36^. For quantification of the tracer accumulation in the brain tissue, 20 µm standard tissue sections of homogenized chicken liver, incubated with defined *cis*-F-d-proline concentrations (1.5 MBq, 0.8 MBq, 0.4 MBq, 0.2 MBq and 0.04 MBq) were sliced and co-exposed overnight with the mouse brain samples to a Fuji BAS phosphor imaging plate TR2025 (Fuji, Japan) in a light-impermeable photo cassette. For evaluation, phosphor imaging plates were scanned using a BAS Reader 5000 (Fuji, Japan), recorded with a width of 4, sensitivity 10,000 and resolution of 25 µm. Finally, monochromatic pictures were evaluated with the Advanced Image and Data Analyzer Software AIDA v4.50 (Raytest Isotopenmessgeräte GmbH, Germany). Circular regions of interest (ROIs) with 0.4 mm diameter were defined in areas of the hippocampus, thalamus, striatum and brainstem. Region calibration of ^18^F tracer accumulation in the standard tissue sections and subtraction of background uptake was performed additionally. Tracer accumulation was finally evaluated as the mean standardized uptake value SUV ± SEM for every 10th section between 2.44 mm to 1.44 mm lateral to Bregma for hippocampus, striatum and brainstem (number of slices, *n* = *6* per animal) and thalamus between 1.84 and 1.44 mm (number of slices, *n* = *3* per animal). The standardized uptake value was calculated as follows:

1 $$SUV= \frac{r}{N(t)}\cdot w$$

Basic SUV was calculated as the quotient of radioactivity concentration [MBq·g^−1^] measured within the ROI of the autoradiogram, $N(t)$ is the decay corrected amount of injected radiolabeled *cis*-F-d-proline (MBq), and $w$ the weight of the mouse (g). Due to decay, values were corrected for the time of tracer injection into the mice:

2 $$N(t)={N}_{0}\cdot {e}^{-\lambda t}$$

with ${N}_{0}$ being the radioactivity at the time point of tracer injection (MBq), $\lambda$ the decay constant of [^18^F] in (1·min^−1^) and the interval $t$ as time between injection and final measured radioactivity in (min). Since no significant differences were found in the *cis*-F-d-proline SUV in the brainstem of all groups, this brain region was considered a suitable reference region (Suppl. Table 4). Therefore, a ratio SUV (SUVR_(Brainstem)_), with the brainstem as reference region, was evaluated to compare tracer accumulation in the ROIs, normalized to the ^18^F background signal of the brain tissue.

### Immunofluorescence staining

For brain extraction, mice were deeply anaesthetised with isoflurane (Isothesia 1000 mg/g liquid, Henry Schein Vet GmbH, Germany) and hemispheres separated for further preparation. 20 µm thick sections were fixed with either 4% formaldehyde or ice-cold (∼4 °C) acetone for 10 min. Antigen retrieval with 70% formic acid and 15 min incubation was required for CD11b staining. Between incubations, slides were washed three times with different buffer solutions for 5 min, respectively with Tris-buffered saline (TBS), if required containing Triton-X. Afterwards, incubation of the sections with the initial primary antibodies (Anti-GFAP rabbit polyclonal, Dako GmbH, Germany, Mouse Anti-NeuN IgG1, clone A60 Merck KGaA, Germany, Anti CD11b rabbit monoclonal, Abcam plc, United Kingdom) in dilution buffers either containing normal goat serum and/or bovine serum albumin (BSA), were incubated overnight at 4 °C (NeuN, GFAP) and RT (CD11b) in a humid and dark chamber. Sections were washed thoroughly three times with desired buffers for 5 min, followed by incubation with the secondary antibodies (Alexa Fluor 488 goat anti-mouse IgG (H + L); Alexa Fluor 488 goat anti-rabbit IgG (H + L), Alexa Fluor 568 goat anti-rabbit IgG (H + L); 2 mg/ml; Thermo Fisher Scientific Inc., Germany), diluted 1:500 in the appropriate dilution buffers and incubated for two hours at RT in a humid, dark chamber. After counterstaining with 4′,6-diamidino-2-phenylindole dihydrochloride (DAPI), slides were washed for 5 min each. Microscopy slides were mounted with Fluorescence Mounting Medium (Dako GmbH, Germany) and dried over night at RT. Images were recorded with a Leica LMD 6000 microscope (Leica Biosciences LAS 4.0, Leica). Motor cortex (MC) and brainstem (BS) images were recorded with a Zeiss Lumar V12 SteREO microscope (AxioVision 6.4 RE, Zeiss). Quantification was performed with the open-source cell image analysis software CellProfiler available at http://www.cellprofiler.org (Cell Image analysis software, version 3.1.8)^64^. Image analysis pipelines (provided serial analysis algorithms) were constructed to count NeuN positive cells and GFAP and CD11b immunoreactive (IR) areas from the average of 3–5 slices of each mouse (*WT n* = *5*, *ClC3*^*td/td*^* n* = *6, ClC3*^*−/−*^* n* = *6).* WT and ClC-3^*td/td*^ slices (average of 2–3) were analyzed regarding reactive astrocytes with GFAP in the motor cortex and brainstem (*WT n* = *5, ClC-3*^*td/td*^ and *-ClC3*^*−/−*^* n* = *6*). Image acquisition to guarantee colour-blind safety was performed using Fiji (ImageJ 1.54f)^65^.

### RNA isolation and qRT-PCR

Hippocampus and thalamus were collected from P60 WT C57BL/6 mouse strain. RNA isolation and qPCR were performed as previously described^66^. Briefly, tissue samples were homogenized in TRIzol, mixed with chloroform, and centrifuged to separate the RNA-containing aqueous phase. RNA was precipitated with isopropanol, washed with ethanol, and then resuspended in RNase-free water. Genomic DNA was removed using DNase I (Thermo Fisher Scientific, Ref. 18068015). For quantitative RT-PCR (qRT-PCR), cDNA was synthesized from 1 µg of total RNA. Gene-specific primers for ClC-3 and ClC-4 were designed to target sequences in the coding region of the mouse *Clcn3* and *Clcn4* genes that allow amplification of all the existing splice variants; for ClC-3 forward 5ʹ- CCTCTTATGGCTGCAGTAATGACC-3ʹ, reverse 5ʹ- GCACTGCCTCAGACCAAGCTT-3ʹ; ClC-4 forward 5ʹ-GACGTGGGGACCTACGAGGACTTCC-3ʹ, reverse 5ʹ- CACTCAAAATAGTCTTTATCTCGGGTATGCC-3ʹ. For the reference genes 18S (Genebank No. NR_003278.3), forward 5ʹ-CGCCGCTAGAGGTGAAATTCTTG-3ʹ, reverse 5ʹ- GTGGCTGAACGCCACTTGTCC-3ʹ. The qRT-PCR was performed using the Maxima SYBER Green qPCR Master Mix (Thermo Fisher Scientific, Ref. K0251) and run on a BIORAD instrument (thermal cycle C1000 Touch, CFX96 real-time system). The transcription levels of ClC-3 and ClC-4 were analysed and compared to that of the reference gene (18S). The data were analyzed according to the CFX manager (Bio-Rad) recommended protocols with single base line threshold auto calculated at 242 reference fluorescent units (RFU).

### Immunoblotting

Hippocampal and thalamus tissue was dissociated and homogenized in RIPA buffer (150 mM NaCl; 50 mM Tris–HCl, pH 8.0; 5 mM EDTA; 1% NP-40; 0.5% sodium deoxycholate; 0.1% SDS, pH 8.0) supplemented with protease inhibitors (Roche, #11836145001) by passing the tissue 5 times though a 200 µL pipette tip followed by 5 passes through a 27G needle. Cells were lysed for 30 min on ice and insoluble material was removed by centrifugation at 16,000×*g* and 4 °C for 15 min. Supernatants were mixed with 4xLDS buffer (Invitrogen, NP0007) supplemented with 400 mM DTT, and heated to 70 °C for 15 min. After centrifugation for 16,000×*g* for 1 min, equal protein amounts (Pierce BCA Protein Assay Kit; Thermo Fischer, #23225) were separated by SDS-PAGE (10% acrylamide gels, Tris/Glycine system) and proteins were transferred to nitrocellulose membrane (Amersham, #10600002). After blocking unspecific binding sites with blocking solution (5% milk powder in Tris-buffered saline supplemented with 0.05% Tween20 (TBS-T)), membranes were probed with specific rabbit antibodies against ClC-3^20^ (kindly provided by Thomas Jentsch, FMP Berlin) and ClC-4^67^ (kindly provided by Thomas Jentsch, FMP Berlin), and mouse anti-GAPDH (abcam, #ab8245) diluted 1:1000 in blocking solution, followed by incubation with HRP-labeled secondary antibodies (abcam, #ab205722 and #ab205724, respectively) diluted 1:5000 in blocking solution. Antibody binding was visualized by enhanced chemiluminescence reaction (Thermo Fischer, #34580) and imaged with a chemiluminescent imaging system (azure biosystems, #500Q).

### Electrophysiology

#### Slice preparation

*Preparation of acute brain slices.* Mice were deeply anaesthetised and subsequently sacrificed. Brains were quickly removed and transferred into ice-cold (∼4 °C) slicing solution (206 mM sucrose; 2.5 mM KCl; 25 mM Glucose; 25 mM NaHCO_3_; 1.25 mM NaH_2_PO_4_; 1 mM CaCl_2_; 3 mM MgCl_2_; 3 mM *myo*-inositol; 2 mM Na-pyruvate; 0.4 mM ascorbic acid). Oxygenation and 7.4 pH levels were maintained with carbogen gas (95% O_2_ and 5% CO_2_). Thalamocortical slices (300 µm in thickness cut from the rostral surface) were prepared using a vibrating blade microtome (Leica VT1200S, Heidelberg, Germany)^68,69^, incubated at 35 °C for ~ 0.5 h in an artificial cerebrospinal fluid (aCSF) (125 mM NaCl, 25 mM d-glucose, 25 mM NaHCO_3_, 2.5 mM KCl, 2 mM CaCl_2_, 1.25 mM NaH_2_PO_4_, 1 mM MgCl_2_) and gradually cooled down to RT before use.

#### Electrophysiological recording

Slices were transferred to the recording chamber and perfused continuously at a speed of ~ 5 ml/min with aCSF bubbled with carbogen gas and maintained at RT. Neurons were visualized under an upright microscope, fitted with 4×/0.13 numerical aperture (NA) and 40× water immersion/0.80 NA objectives (Olympus Deutschland GmbH, Hamburg, Germany). Individual thalamic neurons were identified at 40× magnification under infrared differential inference contrast (IR-DIC) microscopy^70,71^. Patch pipettes (5–7 MΩ) were pulled from thick-wall borosilicate glass capillaries (outer diameter 2.0 mm; inner diameter 1.0 mm) and filled with an internal solution containing: 135 mM K-gluconate, 4 mM KCl, 10 mM HEPES, 10 mM phosphocreatine, 4 mM Mg-ATP, 0.3 mM GTP (pH 7.4, 290–300 mOsm) supplemented with 5 mg/ml biocytin (13.4 mM) to obtain permanent staining for morphological reconstructions after the electrophysiological recordings. The resting membrane potential (V_rest_) was measured immediately after breaking the giga-seal to establish the whole-cell configuration. Membrane potentials were not corrected for a junction potential, which was calculated to be − 15.8 mV^72,73^. The recordings were made using an EPC10 amplifier (HEKA, Lambrecht, Germany). Data were filtered at 2.9 kHz and sampled at 10 kHz using Patch-master software (HEKA), and later analyzed offline using custom-written macros in Igor Pro 6 (Wavemetrics).

#### Electrophysiological data analysis

Resting membrane potential (V_rest_) and the series resistance (R_S_) were measured immediately after establishing the whole-cell configuration. Only neurons with a stable V_rest_ below − 55 mV and a R_S_ of less than 40 MΩ were included for the data analysis to guarantee a high recording quality. Passive membrane properties such as the input resistance (R_in_), membrane time constant(τ_m_), voltage sag, and rheobase current were determined by injecting 1 s rectangular current pulse from − 50 A to + 50 pA with a 10 pA step size into the neurons. When injecting a 1 s 50 pA rectangular current pulse into thalamic neurons at V_rest_ (− 60 mV), rebound action potential (AP) bursts will be generated near the end of current injections due to the activation of low-threshold Ca^2+^ currents^39^. Properties of rebound bursts, such as the number of APs and their duration, were calculated. The AP threshold, half-width, amplitude, latency, and afterhyperpolarization (AHP) amplitude were measured for the first spike elicited by a rheobase current injection. Maximum firing frequency and the slope of the frequency-current (F-I) curve were measured for the spike train during a series of 1 s rectangular current pulse injections from − 20 pA to maximum, in 25 pA increments. Only excitatory neurons were included in our analysis, as they represent the predominant cell population in the VPM (ventral posteriomedial nucleus of the thalamus). Inhibitory neurons are rare in this region (~ 1–4%)^74^. We confirmed the excitatory identity of the recorded neurons based on their electrophysiological properties, including action potential half-width, maximum firing frequency, and rebound bursting behaviour.

#### Histological processing

After electrophysiological recordings, slices containing biocytin-filled neurons were processed using a protocol described previously^75^. Slices were fixed in 4% PFA (12.9 mM) in 100 mM PBS for at least 24 h at 4 °C. To block any endogenous peroxidase activity, slices were treated with 3% H_2_O_2_ (29.4 mM) solution in PBS for about 20 min, rinsed repeatedly using 100 mM PBS and subsequently incubated at RT in 1% avidin-biotinylated horseradish peroxidase (Vector ABC staining kit, Vector Lab. Inc.) containing 0.1% Triton X100 for 1 h. This was followed by chromogenic reaction by adding 0.5 mg/ml (13.4 mM) 3,3diaminobenzidine (DAB; Sigma-Aldrich, USA) until the biocytin-filled neurons with distinct axonal and dendritic branches were clearly visible. Slices were rinsed again with 100 mM PBS, dehydrated slowly for 2–4 h with an increasing ethanol series and cleared in xylene (see Marx, et al.^75^ for details). They were then mounted on gelatinized slides and embedded using Eukitt medium (Otto Kindler GmbH. Germany).

#### Neuronal imaging and reconstruction

Slices containing the biocytin-labelled neurons were imaged at several magnifications of 2.5×, 10×, 20× and 450× with an Olympus BX53 microscope (Olympus, Hamburg, Germany) to obtain images of whole slices and individual neurons. Biocytin-labelled neurons were morphologically reconstructed using Neurolucida® software (MicroBrightField, Williston, VT, USA) on an upright microscope equipped with a motorized stage. An oil-immersion objective at a magnification of 100× was used. The tissue shrinkage was corrected for the three-dimensional neuronal reconstruction using correction factors of 1.1× in the x–y direction and 2.1× in the z direction^75^.

### Statistical analysis

Data are presented as the average mean ± standard error of mean (SEM)*.* Statistical analyses were performed using a non-parametric Wilcoxon-Mann–Whitney two-sample rank test or the Single Measure Parametric Analysis and Repeated Measures (RM) Parametric Analysis of InVivoStat (version 3.7.0) available on https://invivostat.co.uk^76^. Normal distribution was proven with the calculated normal probability plot of InVivoStat. GraphPad PRISM (Version 8.3.1, GraphPad Software, Inc., USA) was utilized for data presentation and for statistical calculations of unpaired students t-test (two-tailed), ordinary and RM two-way ANOVA. Tukey's *post* *hoc *test was used. Values with *P* > 0.05 were considered as not significant (n.s.). Blinding and randomization were established via strict numbering of mice and samples based on their date and order of birth. Only gender was disclosed for reasons of separation in the housing. The analysis of the microscopy images in CellProfiler was predefined by the software in a randomized manner. To achieve a power of minimum 85%, a *priori* analyses with InVivoStat, based on Harrison, DA and Brady^5^, resulted in *n* = 8 per gender for behavioural analyses. However, breeding for longitudinal testing resulted in smaller cohorts because of poor breeding performance.

## Supplementary Information

Supplementary Information.

## Data Availability

The original contributions presented in this study are included in the article and Supplementary material. Further inquiries can be directed to the corresponding authors upon reasonable request.

## References

[1] Izco, M., Carlos, E. & Alvarez-Erviti, L. Impact of endolysosomal dysfunction upon exosomes in neurodegenerative diseases. *Neurobiol. Dis.* **166**, 105651. 10.1016/j.nbd.2022.105651 (2022). 35124191 10.1016/j.nbd.2022.105651

[2] Wang, C., Telpoukhovskaia, M. A., Bahr, B. A., Chen, X. & Gan, L. Endo-lysosomal dysfunction: a converging mechanism in neurodegenerative diseases. *Curr. Opin. Neurobiol.* **48**, 52–58. 10.1016/j.conb.2017.09.005 (2018). 29028540 10.1016/j.conb.2017.09.005

[3] Malik, B. R., Maddison, D. C., Smith, G. A. & Peters, O. M. Autophagic and endo-lysosomal dysfunction in neurodegenerative disease. *Mol. Brain* **12**, 100. 10.1186/s13041-019-0504-x (2019). 31783880 10.1186/s13041-019-0504-x PMC6884906

[4] Giovedi, S., Ravanelli, M. M., Parisi, B., Bettegazzi, B. & Guarnieri, F. C. Dysfunctional autophagy and endolysosomal system in neurodegenerative diseases: relevance and therapeutic options. *Front. Cell Neurosci.* **14**, 602116. 10.3389/fncel.2020.602116 (2020). 33390907 10.3389/fncel.2020.602116 PMC7773602

[5] Jefferies, K. C., Cipriano, D. J. & Forgac, M. Function, structure and regulation of the vacuolar (H+)-ATPases. *Arch. Biochem. Biophys.* **476**, 33–42. 10.1016/j.abb.2008.03.025 (2008). 18406336 10.1016/j.abb.2008.03.025 PMC2543942

[6] Grabe, M. & Oster, G. Regulation of organelle acidity. *J. Gen. Physiol.* **117**, 329–344. 10.1085/jgp.117.4.329 (2001). 11279253 10.1085/jgp.117.4.329 PMC2217256

[7] Stauber, T. & Jentsch, T. J. Chloride in vesicular trafficking and function. *Annu. Rev. Physiol.* **75**, 453–477. 10.1146/annurev-physiol-030212-183702 (2013). 23092411 10.1146/annurev-physiol-030212-183702

[8] Guzman, R. E., Bungert-Plumke, S., Franzen, A. & Fahlke, C. Preferential association with ClC-3 permits sorting of ClC-4 into endosomal compartments. *J. Biol. Chem.* **292**, 19055–19065 (2017). 28972156 10.1074/jbc.M117.801951 PMC5704486

[9] Guzman, R. E., Miranda-Laferte, E., Franzen, A. & Fahlke, C. Neuronal ClC-3 splice variants differ in subcellular localizations, but mediate identical transport functions. *J. Biol. Chem.* **290**, 25851–25862 (2015). 26342074 10.1074/jbc.M115.668186 PMC4646242

[10] Jentsch, T. J. & Pusch, M. CLC chloride channels and transporters: structure, function, physiology, and disease. *Physiol. Rev.* **98**, 1493–1590. 10.1152/physrev.00047.2017 (2018). 29845874 10.1152/physrev.00047.2017

[11] Sahly, A. N. et al. Genotype-phenotype correlation in CLCN4-related developmental and epileptic encephalopathy. *Hum. Genet.*10.1007/s00439-024-02668-z (2024). 10.1007/s00439-024-02668-z 38578438

[12] Palmer, E. E. et al. Functional and clinical studies reveal pathophysiological complexity of CLCN4-related neurodevelopmental condition. *Mol. Psychiatry*10.1038/s41380-022-01852-9 (2022). 10.1038/s41380-022-01852-9 PMC9908558 36385166

[13] Duncan, A. R. et al. Unique variants in CLCN3, encoding an endosomal anion/proton exchanger, underlie a spectrum of neurodevelopmental disorders. *Am. J. Hum. Genet.* **108**, 1450–1465. 10.1016/j.ajhg.2021.06.003 (2021). 34186028 10.1016/j.ajhg.2021.06.003 PMC8387284

[14] Palmer, E. E. et al. De novo and inherited mutations in the X-linked gene CLCN4 are associated with syndromic intellectual disability and behavior and seizure disorders in males and females. *Mol. Psychiatry* **23**, 222–230. 10.1038/mp.2016.135 (2018). 27550844 10.1038/mp.2016.135 PMC5794876

[15] Nakashima, M. et al. De novo CLCN3 variants affecting Gly327 cause severe neurodevelopmental syndrome with brain structural abnormalities. *J. Hum. Genet.*10.1038/s10038-022-01106-0 (2022). 10.1038/s10038-022-01106-0 PMC12327483 36536096

[16] He, H. et al. The molecular and phenotypic spectrum of CLCN4-related epilepsy. *Epilepsia* **62**, 1401–1415. 10.1111/epi.16906 (2021). 33951195 10.1111/epi.16906

[17] Bose, S., He, H. & Stauber, T. Neurodegeneration upon dysfunction of endosomal/lysosomal CLC chloride transporters. *Front. Cell Dev. Biol.* **9**, 639231. 10.3389/fcell.2021.639231 (2021). 33708769 10.3389/fcell.2021.639231 PMC7940362

[18] Dickerson, L. W. et al. Altered GABAergic function accompanies hippocampal degeneration in mice lacking ClC-3 voltage-gated chloride channels. *Brain Res.* **958**, 227–250 (2002). 12470859 10.1016/s0006-8993(02)03519-9

[19] Yoshikawa, M. et al. CLC-3 deficiency leads to phenotypes similar to human neuronal ceroid lipofuscinosis. *Genes Cells* **7**, 597–605 (2002). 12059962 10.1046/j.1365-2443.2002.00539.x

[20] Stobrawa, S. M. et al. Disruption of ClC-3, a chloride channel expressed on synaptic vesicles, leads to a loss of the hippocampus. *Neuron* **29**, 185–196. 10.1016/s0896-6273(01)00189-1 (2001). 11182090 10.1016/s0896-6273(01)00189-1

[21] Sierra-Marquez, J. et al. ClC-3 regulates the excitability of nociceptive neurons and is involved in inflammatory processes within the spinal sensory pathway. *Front. Cell Neurosci.* **16**, 920075. 10.3389/fncel.2022.920075 (2022). 37124866 10.3389/fncel.2022.920075 PMC10134905

[22] Rickheit, G. et al. Role of ClC-5 in renal endocytosis is unique among ClC exchangers and does not require PY-motif-dependent ubiquitylation. *J Biol Chem* **285**, 17595–17603. 10.1074/jbc.M110.115600 (2010). 20351103 10.1074/jbc.M110.115600 PMC2878524

[23] Veeramah, K. R. et al. Exome sequencing reveals new causal mutations in children with epileptic encephalopathies. *Epilepsia* **54**, 1270–1281. 10.1111/epi.12201 (2013). 23647072 10.1111/epi.12201 PMC3700577

[24] Xu, X. et al. Novel CLCN4 variant associated with syndromic X-linked intellectual disability in a Chinese girl: a case report. *BMC Pediatr.* **21**, 384. 10.1186/s12887-021-02860-4 (2021). 34479510 10.1186/s12887-021-02860-4 PMC8414764

[25] Rossi, J. et al. Developmental and epileptic encephalopathy in a young Italian woman with a de novo missense variant in the CLCN4 gene: A case report. *Brain Dev.*10.1016/j.braindev.2023.05.004 (2023). 10.1016/j.braindev.2023.05.004 37271660

[26] Rugarli, E. I. et al. Different chromosomal localization of the Clcn4 gene in Mus spretus and C57BL/6J mice. *Nat. Genet.* **10**, 466–471. 10.1038/ng0895-466 (1995). 7670496 10.1038/ng0895-466

[27] Palmer, S., Perry, J. & Ashworth, A. A contravention of Ohno’s law in mice. *Nat. Genet.* **10**, 472–476. 10.1038/ng0895-472 (1995). 7670497 10.1038/ng0895-472

[28] Stauber, T. & Jentsch, T. J. Sorting motifs of the endosomal/lysosomal CLC chloride transporters. *J. Biol. Chem.* **285**, 34537–34548. 10.1074/jbc.M110.162545 (2010). 20817731 10.1074/jbc.M110.162545 PMC2966069

[29] Weinert, S. et al. Uncoupling endosomal CLC chloride/proton exchange causes severe neurodegeneration. *EMBO J.* **39**, e103358. 10.15252/embj.2019103358 (2020). 32118314 10.15252/embj.2019103358 PMC7196918

[30] Weinert, S. et al. Uncoupling endosomal CLC chloride/proton exchange causes severe neurodegeneration. *Embo J*. e103358 (2020). 10.15252/embj.2019103358 PMC7196918 32118314

[31] Langen, K.-J. et al. Detection of secondary thalamic degeneration after cortical infarction using cis-4-18F-fluoro-D-proline. *J. Nucl. Med.* **48**, 1482–1491 (2007). 17704244 10.2967/jnumed.107.041699

[32] Geisler, S. et al. Detection of remote neuronal reactions in the Thalamus and Hippocampus induced by rat glioma using the PET tracer cis-4-[⁸F]fluoro-D-proline. *J. Cereb. Blood Flow Metab.* **33**, 724–731 (2013). 23385199 10.1038/jcbfm.2013.8 PMC3652687

[33] Guzman, R. E., Grieschat, M., Fahlke, C. & Alekov, A. K. ClC-3 is an intracellular chloride/proton exchanger with large voltage-dependent nonlinear capacitance. *ACS Chem. Neurosci.* **4**, 994–1003. 10.1021/cn400032z (2013). 23509947 10.1021/cn400032z PMC3689194

[34] Kasper, D. et al. Loss of the chloride channel ClC-7 leads to lysosomal storage disease and neurodegeneration. *Embo J.* **24**, 1079–1091. 10.1038/sj.emboj.7600576 (2005). 15706348 10.1038/sj.emboj.7600576 PMC554126

[35] Pressey, S. N. et al. Distinct neuropathologic phenotypes after disrupting the chloride transport proteins ClC-6 or ClC-7/Ostm1. *J. Neuropathol. Exp. Neurol.* **69**, 1228–1246. 10.1097/NEN.0b013e3181ffe742 (2010). 21107136 10.1097/NEN.0b013e3181ffe742

[36] Paxinos, G. & Franklin, K. B. J. *The Mouse Brain in Stereotaxic Coordinates* (Academic Press, 2001).

[37] Hermann, D. M., Mies, G., Hata, R. & Hossmann, K. A. Microglial and astrocytic reactions prior to onset of thalamic cell death after traumatic lesion of the rat sensorimotor cortex. *Acta Neuropathol.* **99**, 147–153. 10.1007/pl00007418 (2000). 10672321 10.1007/pl00007418

[38] Roselli, F. & Caroni, P. From intrinsic firing properties to selective neuronal vulnerability in neurodegenerative diseases. *Neuron* **85**, 901–910. 10.1016/j.neuron.2014.12.063 (2015). 25741719 10.1016/j.neuron.2014.12.063

[39] Llinas, R. & Jahnsen, H. Electrophysiology of mammalian thalamic neurones in vitro. *Nature* **297**, 406–408. 10.1038/297406a0 (1982). 7078650 10.1038/297406a0

[40] Siskova, Z. et al. Dendritic structural degeneration is functionally linked to cellular hyperexcitability in a mouse model of Alzheimer’s disease. *Neuron* **84**, 1023–1033. 10.1016/j.neuron.2014.10.024 (2014). 25456500 10.1016/j.neuron.2014.10.024

[41] Masi, A., Narducci, R. & Mannaioni, G. Harnessing ionic mechanisms to achieve disease modification in neurodegenerative disorders. *Pharmacol. Res.* **147**, 104343. 10.1016/j.phrs.2019.104343 (2019). 31279830 10.1016/j.phrs.2019.104343

[42] Zhao, P., Waxman, S. G. & Hains, B. C. Sodium channel expression in the ventral posterolateral nucleus of the thalamus after peripheral nerve injury. *Mol. Pain* **2**, 27. 10.1186/1744-8069-2-27 (2006). 16916452 10.1186/1744-8069-2-27 PMC1563449

[43] Platkiewicz, J. & Brette, R. A threshold equation for action potential initiation. *PLoS Comput. Biol.* **6**, e1000850. 10.1371/journal.pcbi.1000850 (2010). 20628619 10.1371/journal.pcbi.1000850 PMC2900290

[44] Cerina, M. et al. Thalamic Kv 7 channels: pharmacological properties and activity control during noxious signal processing. *Br. J. Pharmacol.* **172**, 3126–3140. 10.1111/bph.13113 (2015). 25684311 10.1111/bph.13113 PMC4459028

[45] Contet, C., Goulding, S. P., Kuljis, D. A. & Barth, A. L. BK channels in the central nervous system. *Int. Rev. Neurobiol.* **128**, 281–342. 10.1016/bs.irn.2016.04.001 (2016). 27238267 10.1016/bs.irn.2016.04.001 PMC4902275

[46] DosSantos, M. F., Filha, L. G. A., Verissimo, C. P., Sanz, C. K. & Gazerani, P. Presence of small-conductance calcium-activated potassium (SK) channels in the central and peripheral nervous systems and their role in health and disease. *J. Integr. Neurosci.* **22**, 69. 10.31083/j.jin2203069 (2023). 37258451 10.31083/j.jin2203069

[47] Brown, D. A. & Passmore, G. M. Neural KCNQ (Kv7) channels. *Br. J. Pharmacol.* **156**, 1185–1195. 10.1111/j.1476-5381.2009.00111.x (2009). 19298256 10.1111/j.1476-5381.2009.00111.x PMC2697739

[48] Qi, G. et al. Endosomal 2Cl-/H+ exchangers regulate neuronal excitability by tuning Kv7/KCNQ channel density. *Brain*10.1093/brain/awaf243 (2025). 10.1093/brain/awaf243 PMC12677024 40605603

[49] Sierra-Marquez, J. et al. ClC-3 regulates the excitability of nociceptive neurons and is involved in inflammatory processes within the spinal sensory pathway. *Front. Cell. Neurosci.*10.3389/fncel.2022.920075 (2022). 10.3389/fncel.2022.920075 PMC10134905 37124866

[50] Mahajan, K. R., Nakamura, K., Cohen, J. A., Trapp, B. D. & Ontaneda, D. Intrinsic and extrinsic mechanisms of thalamic pathology in multiple sclerosis. *Ann. Neurol.* **88**, 81–92. 10.1002/ana.25743 (2020). 32286701 10.1002/ana.25743 PMC8291218

[51] Kipp, M. et al. Thalamus pathology in multiple sclerosis: from biology to clinical application. *Cell Mol. Life Sci.* **72**, 1127–1147. 10.1007/s00018-014-1787-9 (2015). 25417212 10.1007/s00018-014-1787-9 PMC11113280

[52] Ab Aziz, C. B. & Ahmad, A. H. The role of the thalamus in modulating pain. *Malays. J. Med. Sci.* **13**, 11–18 (2006). 22589599 PMC3349479

[53] Hanada, T. et al. Development and pharmacological verification of a new mouse model of central post-stroke pain. *Neurosci. Res.* **78**, 72–80. 10.1016/j.neures.2013.09.005 (2014). 24055601 10.1016/j.neures.2013.09.005

[54] Allen, A. E., Procyk, C. A., Brown, T. M. & Lucas, R. J. Convergence of visual and whisker responses in the primary somatosensory thalamus (ventral posterior medial region) of the mouse. *J. Physiol.* **595**, 865–881. 10.1113/JP272791 (2017). 27501052 10.1113/JP272791 PMC5285619

[55] Davidson, S., Truong, H. & Giesler, G. J. Jr. Quantitative analysis of spinothalamic tract neurons in adult and developing mouse. *J. Comp. Neurol.* **518**, 3193–3204. 10.1002/cne.22392 (2010). 20575056 10.1002/cne.22392 PMC2996724

[56] Engel, J. P., Madigan, T. C. & Peterson, G. M. The transneuronal spread phenotype of herpes simplex virus type 1 infection of the mouse hind footpad. *J. Virol.* **71**, 2425–2435. 10.1128/JVI.71.3.2425-2435.1997 (1997). 9032380 10.1128/jvi.71.3.2425-2435.1997 PMC191353

[57] Morello, F. et al. ADHD-like behaviors caused by inactivation of a transcription factor controlling the balance of inhibitory and excitatory neuron development in the mouse anterior brainstem. *Transl. Psychiatry* **10**, 357. 10.1038/s41398-020-01033-8 (2020). 33087695 10.1038/s41398-020-01033-8 PMC7578792

[58] Rogers, D. C. et al. Behavioral and functional analysis of mouse phenotype: SHIRPA, a proposed protocol for comprehensive phenotype assessment. *Mamm. Genome* **8**, 711–713 (1997). 9321461 10.1007/s003359900551

[59] Deacon, R. M. J. Assessing nest building in mice. *Nat. Protoc.* **1**, 1117–1119 (2006). 17406392 10.1038/nprot.2006.170

[60] Ogawa, N., Hirose, Y., Ohara, S., Ono, T. & Watanabe, Y. A simple quantitative bradykinesia test in MPTP-treated mice. *Res. Commun. Chem. Pathol. Pharmacol.* **50**, 435–441 (1985). 3878557

[61] Deacon, R. M. J., Croucher, A. & Rawlins, J. N. P. Hippocampal cytotoxic lesion effects on species-typical behaviours in mice. *Behav. Brain Res.* **132**, 203–213 (2002). 11997150 10.1016/s0166-4328(01)00401-6

[62] Hamacher, K. Synthesis of N.C.A. cis- and trans-4-[18F]fluoro-l-proline, radiotracers for PET-investigation of disordered matrix protein synthesis. *J. Labell. Compds. Radiopharmaceuticals* **42**, 1135–1144 (1999).

[63] Wester, H.-J. et al. Preclinical evaluation of 4-[18F]fluoroprolines: diastereomeric effect on metabolism and uptake in mice. *Nucl. Med. Biol.* **26**, 259–265. 10.1016/S0969-8051(98)00107-3 (1999). 10363796 10.1016/s0969-8051(98)00107-3

[64] Lamprecht, M. R., Sabatini, D. M. & Carpenter, A. E. Cell Profiler: free, versatile software for automated biological image analysis. *Biotechniques* **42**, 71–75 (2007). 17269487 10.2144/000112257

[65] Schindelin, J. et al. Fiji: an open-source platform for biological-image analysis. *Nat. Methods* **9**, 676–682. 10.1038/nmeth.2019 (2012). 22743772 10.1038/nmeth.2019 PMC3855844

[66] Comini, M. et al. CLC anion/proton exchangers regulate secretory vesicle filling and granule exocytosis in chromaffin cells. *J. Neurosci.* **42**, 3080–3095. 10.1523/JNEUROSCI.2439-21.2022 (2022). 35241492 10.1523/JNEUROSCI.2439-21.2022 PMC8994546

[67] Maritzen, T., Keating, D. J., Neagoe, I., Zdebik, A. A. & Jentsch, T. J. Role of the vesicular chloride transporter ClC-3 in neuroendocrine tissue. *J. Neurosci.* **28**, 10587–10598. 10.1523/JNEUROSCI.3750-08.2008 (2008). 18923035 10.1523/JNEUROSCI.3750-08.2008 PMC6671342

[68] Feldmeyer, D., Egger, V., Lubke, J. & Sakmann, B. Reliable synaptic connections between pairs of excitatory layer 4 neurones within a single “barrel” of developing rat somatosensory cortex. *J. Physiol.* **521**(Pt 1), 169–190. 10.1111/j.1469-7793.1999.00169.x (1999). 10562343 10.1111/j.1469-7793.1999.00169.x PMC2269646

[69] Agmon, A. & Connors, B. W. Thalamocortical responses of mouse somatosensory (barrel) cortex in vitro. *Neuroscience* **41**, 365–379. 10.1016/0306-4522(91)90333-j (1991). 1870696 10.1016/0306-4522(91)90333-j

[70] Dodt, H. U. & Zieglgansberger, W. Visualizing unstained neurons in living brain slices by infrared DIC-videomicroscopy. *Brain Res.* **537**, 333–336. 10.1016/0006-8993(90)90380-t (1990). 2085783 10.1016/0006-8993(90)90380-t

[71] Stuart, G. J., Dodt, H. U. & Sakmann, B. Patch-clamp recordings from the soma and dendrites of neurons in brain slices using infrared video microscopy. *Pflugers Arch.* **423**, 511–518. 10.1007/BF00374949 (1993). 8351200 10.1007/BF00374949

[72] Barry, P. H. JPCalc, a software package for calculating liquid junction potential corrections in patch-clamp, intracellular, epithelial and bilayer measurements and for correcting junction potential measurements. *J. Neurosci. Methods* **51**, 107–116. 10.1016/0165-0270(94)90031-0 (1994). 8189746 10.1016/0165-0270(94)90031-0

[73] Barry, P. H. & Lynch, J. W. Liquid junction potentials and small cell effects in patch-clamp analysis. *J. Membr. Biol.* **121**, 101–117. 10.1007/bf01870526 (1991). 1715403 10.1007/BF01870526

[74] Simko, J. & Markram, H. Morphology, physiology and synaptic connectivity of local interneurons in the mouse somatosensory thalamus. *J. Physiol.* **599**, 5085–5101. 10.1113/JP281711 (2021). 34591324 10.1113/JP281711 PMC9298088

[75] Marx, M., Gunter, R. H., Hucko, W., Radnikow, G. & Feldmeyer, D. Improved biocytin labeling and neuronal 3D reconstruction. *Nat. Protoc.* **7**, 394–407. 10.1038/nprot.2011.449 (2012). 22301777 10.1038/nprot.2011.449

[76] Clark, R. A., Shoaib, M., Hewitt, K. N., Stanford, S. C. & Bate, S. T. A comparison of InVivoStat with other statistical software packages for analysis of data generated from animal experiments. *J. Psychopharmacol.* **26**, 1136–1142. 10.1177/0269881111420313 (2012). 22071578 10.1177/0269881111420313

