# Supplementary material for: Disruption of ClC-3-mediated 2Cl − /H + exchange leads to behavioural deficits and thalamic atrophy
Source: Sci Rep. 2025 Sep 29;15:33326. doi: 10.1038/s41598-025-19757-2 (PMC12479943; doi:10.1038/s41598-025-19757-2)
Supplement: Supplementary file 1 — Supplementary Information. [file 41598_2025_19757_MOESM1_ESM.pdf]

# Supplementary Material

# Disruption of ClC-3-mediated 2Cl$^-$/H$^+$ exchange leads to behavioural deficits and thalamic atrophy

Carina Balduin[1], Guanxiao Qi[2], Michael Schöneck[1], Verena Trinkel[1], Sarah Schemmert[3], Gustavo A. Guzman[3], Stefanie Bungert-Plümke[4], Malte Klüssendorf[5], Bernd Neumaier[6,7], Dirk Feldmeyer[2,11], N. Jon Shah[1,8,9,10], Tobias Stauber[5], Karl-Josef Langen[1,12], Raul E. Guzman[4,†*], Antje Willuweit[1,†*]

[†]**These authors contributed equally to this work.**

**Author affiliations:**

1 Institute of Neuroscience and Medicine, INM-4, Forschungszentrum Jülich GmbH, 52425 Jülich, Germany

2 Institute of Neuroscience and Medicine, INM-10, Forschungszentrum Jülich GmbH, 52425 Jülich, Germany

3 Institute of Biological Information Processing, IBI-7, Forschungszentrum Jülich GmbH, 52425 Jülich, Germany

4 Institute of Biological Information Processing, IBI-1, Forschungszentrum Jülich GmbH, 52425 Jülich, Germany

5 Institute for Molecular Medicine, MSH Medical School Hamburg, Hamburg, Germany

6 Institute of Neuroscience and Medicine, INM-5, Forschungszentrum Jülich GmbH, 52425 Jülich, Germany

7 University of Cologne, Faculty of Medicine and University Hospital Cologne, Institute of Radiochemistry and Experimental Molecular Imaging, 50937 Cologne, Germany

8 Institute of Neuroscience and Medicine 11, INM-11, JARA, Forschungszentrum Jülich, Germany.

9 JARA - BRAIN - Translational Medicine, Aachen, Germany.

10 Department of Neurology, RWTH Aachen University, Aachen, Germany

11 Department of Psychiatry, Psychotherapy and Psychosomatics, RWTH Aachen University, 52062 Aachen, Germany

12 Department of Nuclear Medicine, RWTH Aachen University, 52062 Aachen, Germany

* Corresponding authors:

Antje Willuweit

Institute of Neuroscience and Medicine INM-4

Forschungszentrum Jülich GmbH

52425 Jülich

Germany

E-Mail: a.willuweit@fz-juelich.de

&

Raul E. Guzman

Institute of Biological Information Processing IBI-1

Forschungszentrum Jülich GmbH

52425 Jülich

Germany

E-Mail: r.guzman@fz-juelich.de

# Supplementary materials and methods

## Animals

By crossing heterozygous *Clcn3*$^{+/td}$, females and males of either genotype were generated (WT; heterozygous *Clcn3*$^{+/td}$; and homozygous *Clcn3*$^{td/td}$). WT and *Clcn3*$^{-/-}$ mice[1] (kindly provided by Thomas Jentsch, FMP Berlin) were used as controls. Ear biopsies were collected from the mice at the age of 16 days, and the genotype was identified with a specific PCR-based genotyping protocol using the KAPA Mouse Genotyping Kit (Kapa Biosystems/Roche, KK-7302, Wilmington, MA, United States), according to the manufacturer's instructions. The following set of primers was used; forward 5'-CACGGGATCACAGTAGTGAAAGG-3' and reverse 5'-CGCTGCAGTCCATTAAACAGTTTC-3'; fragments 332 bp and 250 bp for mutant and WT were expected.

### Behavioural test setup

#### SHIRPA phenotype assessment

Phenotype assessment was analysed based on the primary behavioural screen of the SHIRPA test battery described by Rogers et al.[2] Observations were scored in several subtests, investigating reflexes, sensory perception and motor coordination. Mice were individually observed in their home cage or in the arena (B19.5 cm x H55 cm x W33 cm) in the following subtests: restlessness, apathy, grooming, stereotyped behaviour, convulsions, abnormal body carriage, alertness, abnormal gait, clasping, hanging wire, startle response, touch response, pinna reflex, cornea reflex, whisker touch response, pain response. Behaviour was evaluated with defined scores: 0, normal reaction; 1, eventually changed; 2, changed; 3, extremely changed/ no reaction. The SHIRPA score was the result of the summed scores of a mouse.

#### Pole test

Basal ganglia-associated movement dysfunctions were evaluated using the modified pole test as previously described[3] . A vertical pole 50 cm high with a ridged surface was placed into an arena (B19 cm x H38 cm x W60 cm) and covered with 5 cm of bedding material. Mice were taken by

their tail to the top of the pole, and their movement downwards was rated: 0, running continuously; 1, partly running, then slipping or jumping; 2, slipping; 3, not able to move/ fall from the top. The procedure was repeated three times with a 15-minute intertrial interval. The summed score was used for evaluation.

**Nesting test**

The nesting test was performed using provided materials to analyze the rodent's natural behaviour of nest construction to protect their litters and to preserve heat[4]. For instance, lesions in hippocampal regions could result in the loss of species-typical behaviour. Thus, nesting competence can provide insights into both motor coordination and cognitive performance[5]. Mice were housed separately overnight with food and water ad libitum. A cotton "Nestlet" was offered for environmental enrichment. After 16 h, the nests and the condition of residues from the Nestlets were evaluated by two independent experimenters with the modified 5-point-scoring system proposed by Robert M.J. Deacon: 1 >90 % undamaged; 2, 50 to 90 % cotton shredded in the cage; 3, <50 % intact, no obvious nest; 4, 100 % shredded, flat nest without walls; 5, 100 % shredded, perfect surrounding nest.

**Open field test**

The anxiety-related behaviour, explorative behaviour and locomotion of *Clcn3*$^{td/td}$ mice were analyzed in the open field test. Mice were placed in the right corner of a square-shaped arena (L44 cm x W44 cm x H40 cm). Lighting was maintained at 400 to 500 lux during the experiments. Mice could move freely for 20 min in the arena, after which time the arena was virtually divided into a border (11 cm from the wall) and a centre zone (22 cm x 22 cm middle part). Video tracking software, EthoVision XT (Noldus Information Technologies, Wageningen, Netherlands), was used for the evaluation of the distance moved in the zones (cm), the time spent in the arena zones (s) and the velocity (cm·s−1).

# Cellular analyses

## Immunofluorescence staining

Sections were thawed at room temperature (RT) and fixed with either 4 % formaldehyde or ice-cold (~4°C) acetone for 10 min. Antigen retrieval with 70 % formic acid and 15 min incubation was required for CD11b staining. Between incubations, slides were washed three times with fresh buffer solutions for 5 min with Tris-buffered saline (TBS) containing Triton-X. Afterwards, the sections with the initial primary antibodies (Anti-GFAP rabbit polyclonal, Dako GmbH, Germany, Mouse Anti-NeuN IgG1, clone A60 Merck KGaA, Germany, Anti CD11b rabbit monoclonal, Abcam plc, United Kingdom) in dilution buffers containing either normal goat serum and/or bovine serum albumin (BSA) were incubated overnight at 4°C (NeuN, GFAP) and RT (CD11b) in a dark, humid chamber. Sections were washed thoroughly three times with the desired buffers for 5 min, followed by incubation with the secondary antibodies diluted 1:500 in the appropriate dilution buffers and incubated for two hours at RT in a humid, dark chamber: Alexa Fluor 488 goat anti-mouse IgG (H+L); Alexa Fluor 488 goat anti-rabbit IgG (H+L), Alexa Fluor 568 goat anti-rabbit IgG (H+L); 2 mg/ml; Thermo Fisher Scientific Inc., Germany). After counterstaining with 4′,6-diamidino-2-phenylindole dihydrochloride (DAPI), slides were washed for 5 min each. Microscopy slides were mounted with a fluorescence mounting medium (Dako GmbH, Germany) and dried overnight at RT. Images were recorded with a Leica LMD 6000 microscope (Leica Biosciences LAS 4.0, Leica). Motor cortex (MC) and brainstem (BS) images were recorded with a Zeiss Lumar V12 SteREO microscope (AxioVision 6.4 RE, Zeiss). Quantification was performed with the open-source cell image analysis software CellProfiler, which is available at http://www.cellprofiler.org (Cell Image analysis software, version 3.1.8)[6]. Image analysis pipelines (provided serial analysis algorithms) were constructed to count NeuN positive cells and GFAP and CD11b immunoreactive (IR) areas from the average of 3-5 slices of each mouse (*WT n=5*, $ClC3^{td/td}$ *n=6,* $ClC3^{-/-}$ *n=6).* WT and ClC-$3^{td/td}$ slices (average of 2-3) were analyzed regarding reactive astrocytes with GFAP in the motor cortex and brainstem (*WT n=5,* $ClC\text{-}3^{td/td}$ and -$ClC3^{-/-}$ *n=6*). Image acquisition To guarantee colour-blind safety, image acquisition was performed with Fiji ImageJ 1.54f [7].

**RNA isolation and qRT-PCR**

The hippocampus and thalamus were collected from the P60 WT C57BL/6 mouse strain. RNA isolation and qPCR were performed as previously described[8]. Briefly, tissue samples were homogenized in TRIzol, mixed with chloroform, and centrifuged to separate the RNA-containing aqueous phase. The RNA was precipitated with isopropanol, washed with ethanol, and then resuspended in RNase-free water. The genomic DNA was removed using DNase I (Thermo Fisher Scientific, Ref. 18068015). For quantitative RT-PCR (qRT-PCR), cDNA was synthesized from 1 μg of the total RNA. Gene-specific primers for ClC-3 and ClC-4 were designed to target sequences in the coding region of the mouse *Clcn3* and *Clcn4* genes that allow amplification of all the existing splice variants; for ClC-3 forward 5’- CCTCTTATGGCTGCAGTAATGACC-3’, reverse 5’-GCACTGCCTCAGACCAAGCTT-3’; ClC-4 forward 5’-GACGTGGGGACCTACGAGGACTTCC-3’, reverse 5’-CACTCAAAATAGTCTTTATCTCGGGTATGCC-3’. For the reference genes 18S (Genebank No. NR_003278.3), forward 5’-CGCCGCTAGAGGTGAAATTCTTG-3’, reverse 5’-GTGGCTGAACGCCACTTGTCC-3’. The qRT-PCR was performed using the Maxima SYBER Green qPCR Master Mix (Thermo Fisher Scientific, Ref. K0251) and run on a BIORAD instrument (thermal cycle C1000 Touch, CFX96 real-time system). The transcription levels of ClC-3 and ClC-4 were analyzed and compared to that of the reference gene (18S). The data were analyzed according to the CFX manager (Bio-Rad) recommended protocols with a single baseline threshold auto-calculated to 242 reference fluorescent units (RFU).

**Immunoblotting**

Hippocampal and thalamus tissue were dissociated and homogenized in RIPA buffer (150 mM NaCl; 50 mM Tris–HCl, pH 8.0; 5 mM EDTA; 1% NP-40; 0.5% sodium deoxycholate; 0.1% SDS, pH 8.0) supplemented with protease inhibitors (Roche, #11836145001) by passing the tissue five times though a 200 μL pipette tip followed by 5 passes through a 27G needle. Cells were lysed for 30 min on ice, and insoluble material was removed by centrifugation at 16,000 × g and 4°C for 15 min. Supernatants were mixed with 4xLDS buffer (Invitrogen, NP0007) supplemented with 400 mM DTT and heated to 70°C for 15 min. After centrifugation for 16.000 x g for 1 min, equal protein amounts (Pierce BCA Protein Assay Kit; Thermo Fischer, #23225) were separated using

SDS-PAGE (10% acrylamide gels, Tris/Glycine system) and proteins were transferred to nitrocellulose membrane (Amersham, #10600002). After blocking unspecific binding sites with a blocking solution (5% milk powder in Tris-buffered saline supplemented with 0.05% Tween20 (TBS-T)), the membranes were probed with specific rabbit antibodies against ClC-3[1] (kindly provided by Thomas Jentsch, FMP Berlin), ClC-4 [9] ( kindly provided by Thomas Jentsch, FMP Berlin), and mouse anti-GAPDH (abcam, #ab8245) diluted 1:1000 in blocking solution, followed by incubation with HRP-labelled secondary antibodies (abcam, #ab205722 and #ab205724, respectively) diluted 1:5000 in blocking solution. Antibody binding was visualized by enhanced chemiluminescence reaction (Thermo Fischer, #34580) and imaged with a chemiluminescent imaging system (Azure Biosystems, #500Q).

**Electrophysiological data analysis**

Resting membrane potential ($V_{rest}$) and the series resistance ($R_S$) were measured immediately after establishing whole-cell configuration. Only neurons with a stable $V_{rest}$ below −55 mV and an $R_S$ of less than 40 MΩ were included for the data analysis to guarantee a high recording quality. Passive membrane properties, such as the input resistance ($R_{in}$), membrane time constant($\tau_m$), voltage sag, and rheobase current, were determined by injecting 1 s rectangular current pulse from −50 A to +50 pA with a 10 pA step size to the neurons. When injecting a 1 s 50 pA rectangular current pulse into thalamic neurons at $V_{rest}$ (−60 mV), rebound action potential (AP) bursts will be generated near the end of current injections due to the activation of low-threshold $Ca^{2+}$ currents[10]. The properties of rebound bursts, such as the number of APs and their duration, were calculated. The AP threshold, half-width, amplitude, latency, and afterhyperpolarization (AHP) amplitude were measured for the first spike elicited by a rheobase current injection. The maximum firing frequency and the slope of the frequency-current (F-I) curve were measured for the spike train during a series of 1 s rectangular current pulse injections from −20 pA to maximum, in 25 pA increments.

**Histological processing**

After electrophysiological recordings, slices were fixed in 100 mM PBS containing 4 % PFA (12.9 mM) for at least 24 h at 4°C. To block endogenous peroxidase activity, slices were treated

with 3 % $H_2O_2$ (29.4 mM) solution in PBS for about 20 min, rinsed repeatedly using 100 mM PBS and subsequently incubated at RT in 1 % avidin-biotinylated horseradish peroxidase (Vector ABC staining kit, Vector Lab. Inc.) containing 0.1 % Triton X-100 for 1 h. This was followed by a chromogenic reaction by adding 0.5 mg/ml (13.4 mM) 3,3-diaminobenzidine (DAB; Sigma-Aldrich, USA) until the biocytin-filled neurons with distinct axonal and dendritic branches were clearly visible. Slices were rinsed again with 100 mM PBS, dehydrated slowly for 2-4 h with an increasing ethanol series and cleared in xylene (see ref [10] for details). They were then mounted on gelatinized slides and embedded using Eukitt medium (Otto Kindler GmbH, Germany).

# Supplementary results

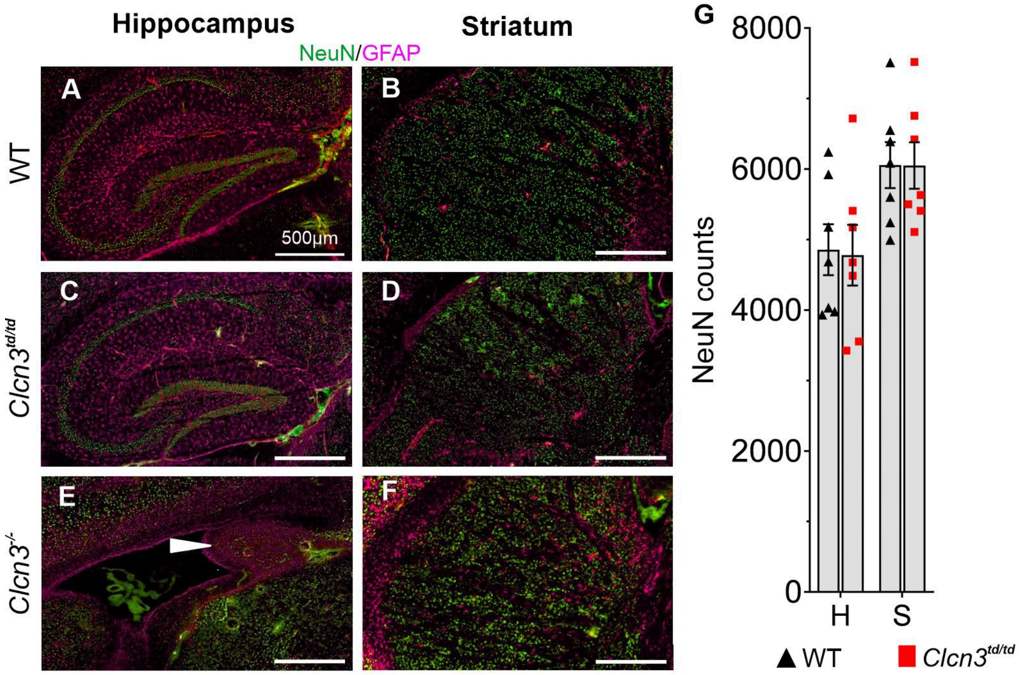


**Supplementary Figure 1. Representative immunofluorescence images of the hippocampus and striatum** in sagittal sections of (A-B) WT, (C-D) *Clcn3*$^{td/td}$ and (E-F) *Clcn3*$^{-/-}$ P70 mice stained against neurons (NeuN, green) and activated astrocytes (GFAP, magenta). Degeneration of the hippocampus formation is only obvious in *Clcn3*$^{-/-}$ mice (hippocampus position, white arrow). The number of NeuN-positive cells is markedly decreased in this area, whereas GFAP-positive activated astrocytes are present in the tissue. (**G**) Analyses of the different brain areas, i.e., the hippocampus (H) and striatum (S), resulted in no significant differences of NeuN-positive cells in *Clcn3*$^{td/td}$ (red squares) mice compared to WT (black up triangles) mice (genotype: $F(1, 24) = 0{,}01304$, $p = 0.91$). WT, n = 7, *Clcn3*$^{td/td}$, n = 7. Due to limited tissue samples *Clcn3*$^{-/-}$

were not analyzed. Data are presented as mean ± SEM averaged from three to five slices per mouse. Statistical calculation was conducted using two-way ANOVA with Fisher's *post hoc* analysis WT vs. *Clcn3*$^{td/td}$.

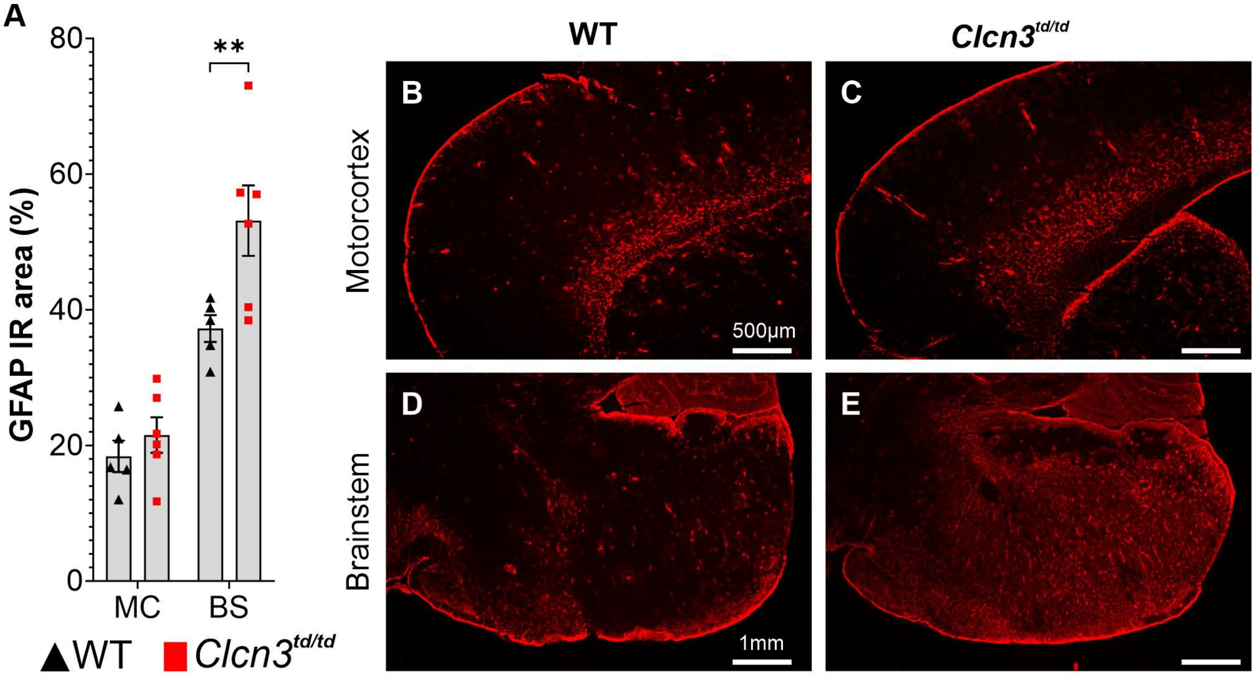


**Supplementary Figure 2. Astrocytosis in the brainstem of *Clcn3*$^{td/td}$ mice**. **(A)** Analyses of the different brain areas, i.e., the motor cortex (MC) and brainstem (BS), resulted in a significantly increased immunoreactive area of reactive astrocytes in the brainstem of *Clcn3*$^{td/td}$ (red squares) mice compared to WT (black up triangles) mice. **(B-E)** Representative example images of the analysed BS and MC from *Clcn3*$^{td/td}$ mice. GFAP-positive reactive astrocytes are shown in red. WT, $n = 5$, *Clcn3*$^{td/td}$, $n = 6$. Data are presented as mean ± SEM averaged from two to three slices per mouse. Statistical calculation was conducted using two-way ANOVA with Fisher's *post hoc* analysis WT vs. *Clcn3*$^{td/td}$. Significant data are marked with asterisks. $^{**}P \leq 0.01$.

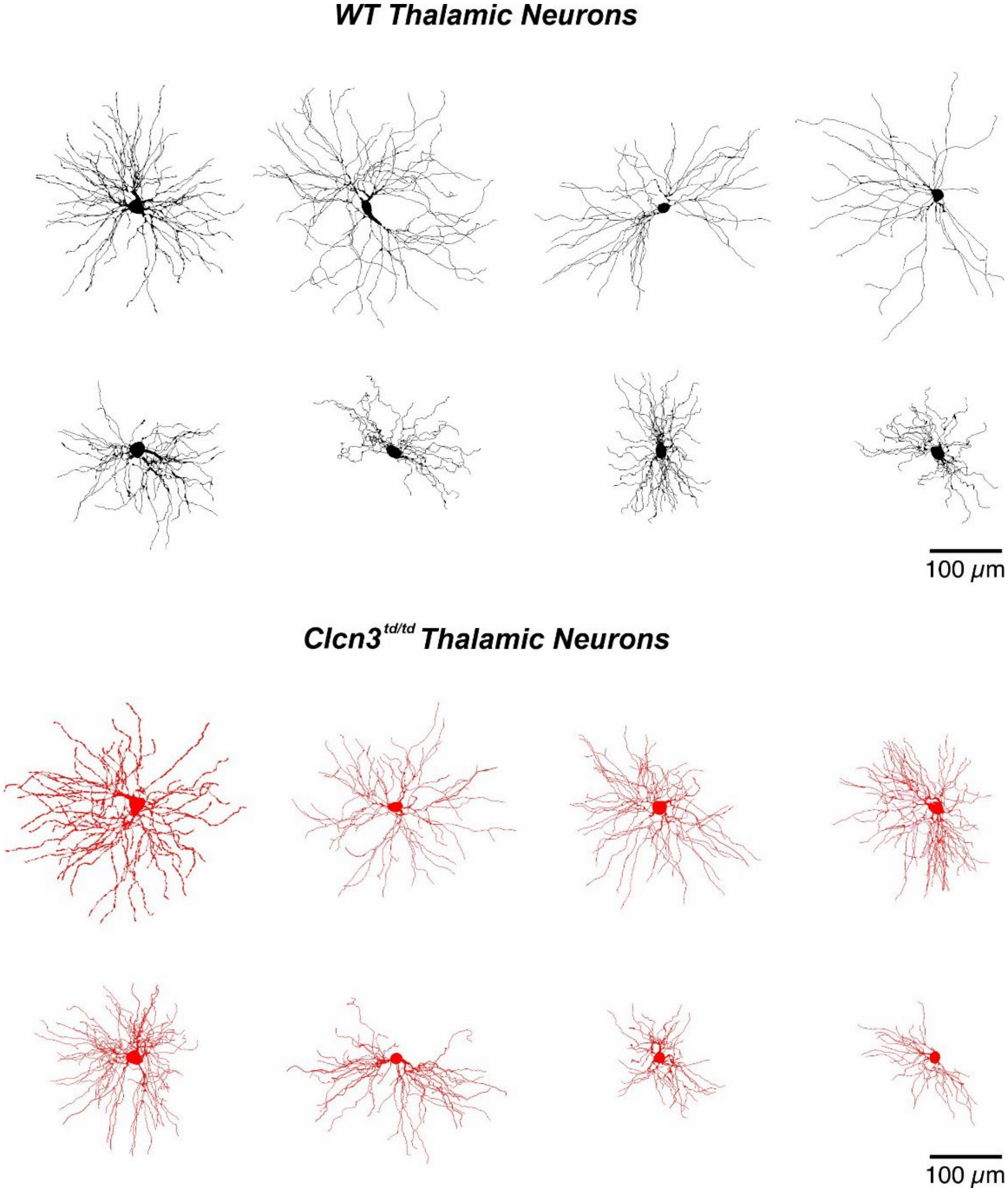


**Supplementary Figure 3. Morphological reconstruction of thalamic neurons in WT and *Clcn3*$^{td/td}$ mice**. Somatodendrites of neurons from WT and *Clcn3*$^{td/td}$ mice are given in black and red, respectively.

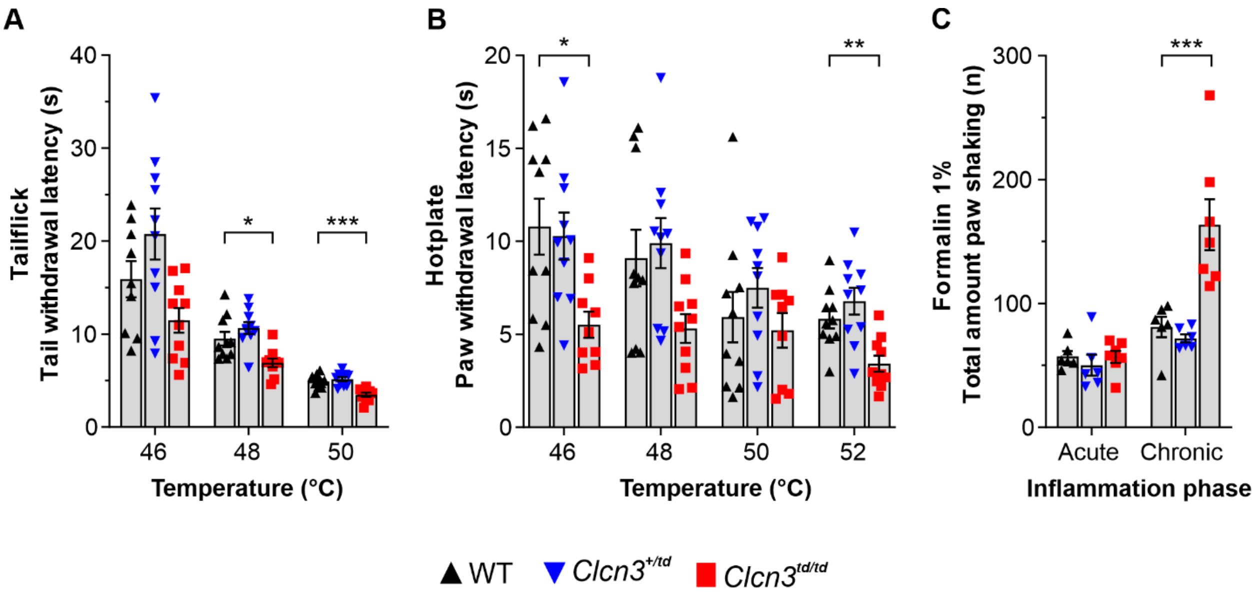


**Supplementary Figure 4. Sensitivity towards thermal and chemical noxious stimuli. (A)** The tail-flick test verified a thermal heat sensitivity for *ClCn3*$^{td/td}$ (red squares) mice at temperatures above 46 °C and an overall trend towards decreased reaction times for *Clcn3*$^{td/td}$ mice against heat stimuli in the **(B)** hotplate withdrawal test, where higher integration of CNS centres is involved to cause a paw lifting. **(C)** The formalin test, where 1% formalin solution was injected into a hind paw to test the mice's reaction to noxious pain stimuli, showed a significantly increased chronic pain reaction for *Clcn3*$^{td/td.}$ mice. Data are presented as mean ± SEM. Statistical calculation was conducted using mixed effect analysis for A) and B) with Dunnett *post hoc* test. WT (black up triangles), $n$ = 10, *Clcn3*$^{+/td}$ (blue down triangles), $n$ = 10; *Clcn3*$^{td/td}$ (red squares), $n$ = 10. A two-way repeated measures ANOVA with Dunnett *post hoc* test was performed for C). WT, $n$ = 6, *Clcn3*$^{+/td}$, $n$ = 6; *Clcn3*$^{td/td}$, $n$ = 7 Significant data are marked with asterisks *$P \leq 0.05$, **$P \leq 0.01$, ***$P \leq 0.001$.

# Supplementary Tables

**Supplementary Table 1.** Comparison of **electrophysiological properties of thalamic neurons from adult WT and *Clcn3*$^{td/td}$ mice.**

| | **WT** ($n$ = 10) | ***Clcn3***$^{td/td}$ ($n$ = 10) | **Statistics** (***p* values** ) |
|---|---|---|---|
| **Passive** | | | |
| $V_{rest}$ (mV) | -61.9 ± 0.9 | -61.2 ± 0.7 | 0.35 |
| $R_{in}$ (MΩ) | 281.0 ± 31.2 | 250.9 ± 35.7 | 0.63 |
| $\tau_m$ (ms) | 34.6 ± 1.8 | 28.2 ± 3.5 | 0.11 |
| Sag (%) | 21.1 ± 5.5 | 14.5 ± 6.3 | 0.10 |
| **Rebound burst** | | | |
| No. of APs per burst | 7.5 ± 0.8 | 5.9 ± 0.8 | 0.20 |
| Burst duration (ms) | 61.0 ± 6.2 | 46.8 ± 5.8 | 0.11 |
| **Single AP** | | | |
| Rheobase current (pA) | 35.0 ± 8.1 | 64.0 ± 16.4 | 0.29 |
| AP threshold (mV) | -42.2 ± 0.7 | -38.9 ± 1.1 | 0.023 |
| AP half-width (ms) | 0.90 ± 0.08 | 0.94 ± 0.04 | 0.80 |
| AP amplitude (mV) | 93.1 ± 2.6 | 108.5 ± 3.3 | 0.002 |
| AP latency (ms) | 153 ± 61 | 189 ± 55 | 0.35 |
| AHP amplitude (mV) | 11.3 ± 0.7 | 16.8 ± 0.9 | 0.0002 |
| *Repetitive firing* | | | |
| Max. firing frequency (Hz) | 44.8 ± 4.9 | 46.9 ± 5.8 | 0.95 |
| Slope of F-I curve (APs/100 pA) | 26.1 ± 3.6 | 26.5 ± 5.2 | 0.57 |

*P* values were calculated using the non-parametric Wilcoxon-Mann-Whitney two-sample rank test. Data were presented as mean ± standard error of the mean (SEM) or the median.

**Supplementary Table 2. Fisher *post hoc* analyses of WT *versus ClCn3*$^{td/td}$ and *ClCn3*$^{-/-}$ mice.**

| | **P20** | **P30** | **P40** | **P60** |
|---|---|---|---|---|
| **Body weight** | | | | |
| WT vs *Clcn3*$^{td/td}$ | p<.01 | p<.0001 | p<.0001 | p<.0001 |
| WT vs *Clcn3*$^{-/-}$ | p<.01 | p<.0001 | p<.0001 | p<.01 |
| **SHIRPA** | | | | |
| WT vs *Clcn3*$^{td/td}$ | p<.001 | p<.05 | p<.001 | p<.0001 |
| WT vs *Clcn3*$^{-/-}$ | p<.0001 | p<.0001 | p<.0001 | p<.0001 |
| **Nesting** | | | | |
| WT vs *Clcn3*$^{td/td}$ | n.a. | n.s. | p<.0001 | p<.0001 |
| WT vs *Clcn3*$^{-/-}$ | n.a. | n.s. | n.s. | p<.0001 |
| **Pole test** | | | | |
| WT vs *Clcn3*$^{td/td}$ | p<.0001 | p<.0001 | p<.0001 | p<.0001 |

*Clcn3*$^{td/td}$, *Clcn3*$^{-/-}$, and WT, *n.a.*, not analysed, *n.s.* not significant.

**Supplementary Table 3. Overview of analysed mice**

| **Experiments** | **Number of mice** | | | | | |
|---|---|---|---|---|---|---|
| | ***Knock-in*** | | | ***Knock-out*** | | |
| | ***Clcn3***$^{+/+}$ | ***Clcn3***$^{+/td}$ | ***Clcn3***$^{td/td}$ | ***Clcn3***$^{+/+}$ | ***Clcn3***$^{+/-}$ | ***Clcn3***$^{-/-}$ |
| **Autoradiography (cross sectional)** | | | | | | |
| P30 (P21 to 31) | 6[a] | — | 6 | 6[a] | — | 5 |
| P70 (P57 to 76) | 10[b] | — | 6 | 10[b] | — | 5 |
| **Histopathological analyses (P57 to 76)** | 10[c] | — | 6 | 10[c] | — | 6 |
| **Electrophysiology (P50 to77)** | 5 | | 5 | | | |
| **Behaviour and motor functions (longitudinal)** | | | | | | |
| Body weight | 12 | 6 | 12 | 11 | 13 | 12 |
| SHIRPA, nesting | 6 | 6 | 7 | 11 | 14 | 11 |
| Open field, pole test, | 6 | 6 | 7 | — | — | — |

[a,b,c] group of WT mice similar for both mouse lines

P(x), postnatal age in days.

**Supplementary Table 4. *cis*-4-[$^{18}$F]-fluoro-D-proline uptake in the brainstem of WT, *Clcn3*$^{td/td}$ and *Clcn3*$^{-/-}$ mice.**

| | | ***cis*-4-[$^{18}$F]-fluoro-D-proline** | | | |
|---|---|---|---|---|---|
| | **Brain area** | **SUV P30** | **Statistics** | **SUV P70** | **Statistics** |
| ***Clcn3***$^{td/td}$ | Brainstem | 0.31 ± 0.12 | | 0.11 ± 0.02 | |
| ***Clcn3***$^{-/-}$ | Brainstem | 0.13 ± 0.02 | P = 0.4084, F(2,14)=0.9552 | 0.13 ± 0.01 | P = 0.5357, F(2,18)=0.6463 |
| ***WT*** | Brainstem | 0.28 ± 0.09 | | 0.14 ± 0.02 | |

P(x)=postnatal age in days, SUV = standardized uptake value. Data are presented as mean ± SEM. Statistics calculated with one-way ANOVA.

# References


1. Stobrawa SM, Breiderhoff T, Takamori S*, et al*. Disruption of ClC-3, a chloride channel expressed on synaptic vesicles, leads to a loss of the hippocampus. *Neuron*. Jan 2001;29(1):185-96. doi:10.1016/s0896-6273(01)00189-1
2. Rogers DC, Fisher EM, Brown SD, Peters J, Hunter AJ, Martin JE. Behavioral and functional analysis of mouse phenotype: SHIRPA, a proposed protocol for comprehensive phenotype assessment. *Mamm Genome*. Oct 1997;8(10):711-3. doi:10.1007/s003359900551
3. Ogawa N, Hirose Y, Ohara S, Ono T, Watanabe Y. A simple quantitative bradykinesia test in MPTP-treated mice. *Res Commun Chem Pathol Pharmacol*. Dec 1985;50(3):435-41.
4. Deacon RM. Assessing nest building in mice. *Nat Protoc*. 2006;1(3):1117-9. doi:10.1038/nprot.2006.170
5. Deacon RM, Croucher A, Rawlins JN. Hippocampal cytotoxic lesion effects on species-typical behaviours in mice. *Behav Brain Res*. May 14 2002;132(2):203-13. doi:10.1016/s0166-4328(01)00401-6
6. Lamprecht MR, Sabatini DM, Carpenter AE. CellProfiler: free, versatile software for automated biological image analysis. *Biotechniques*. 2007;42(1):71-5.
7. Schindelin J, Arganda-Carreras I, Frise E*, et al*. Fiji: an open-source platform for biological-image analysis. *Nature Methods*. 2012/07/01 2012;9(7):676-682. doi:10.1038/nmeth.2019
8. Comini M, Sierra-Marquez J, Guzman G*, et al*. CLC Anion/Proton Exchangers Regulate Secretory Vesicle Filling and Granule Exocytosis in Chromaffin Cells. *J Neurosci*. Apr 13 2022;42(15):3080-3095. doi:10.1523/JNEUROSCI.2439-21.2022
9. Maritzen T, Keating DJ, Neagoe I, Zdebik AA, Jentsch TJ. Role of the vesicular chloride transporter ClC-3 in neuroendocrine tissue. *J Neurosci*. Oct 15 2008;28(42):10587-98. doi:10.1523/JNEUROSCI.3750-08.2008
10. Llinas R, Jahnsen H. Electrophysiology of mammalian thalamic neurones in vitro. *Nature*. Jun 3 1982;297(5865):406-8. doi:10.1038/297406a0
